# Shape Memory Response of Tailored Polylactic Acid/Polycaprolactone Blends: A Validated Constitutive Theoretical Investigation and Sensitivity Analysis

**DOI:** 10.3390/polym18131577

**Published:** 2026-06-25

**Authors:** Giovanni Spinelli, Rosella Guarini, Evgeni Ivanov, Rumiana Kotsilkova, Vittorio Romano

**Affiliations:** 1 Faculty of Transport Sciences and Technologies, University of Study “Giustino Fortunato”, Via Raffaele Delcogliano 12, 82100 Benevento, Italy; 2 Open Laboratory on Experimental Micro and Nano Mechanics, Institute of Mechanics, Bulgarian Academy of Sciences, Acad. G. Bonchev Str., Block 4, 1113 Sofia, Bulgaria; rguarini@imbm.bas.bg (R.G.); ivanov_evgeni@imbm.bas.bg (E.I.); kotsilkova@imbm.bas.bg (R.K.); 3 Department of Industrial Engineering, University of Salerno, Via Giovanni Paolo II, 84084 Fisciano, Italy; vittorioromano2022@gmail.com; 4 Center of Competence for Mechatronics and Clean Technologies “Mechatronics, Innovation, Robotics, Automation and Clean Technologies”—MIRACle, 1113 Sofia, Bulgaria

**Keywords:** green polymer blends, shape memory effect, thermo-mechanical modeling, temperature-dependent elasticity, viscoelastic recovery, smart polymer materials

## Abstract

Shape-memory polymers (SMPs) are gaining significant attention for their ability to recover predefined shapes via external stimuli. Among thermally activated systems, biodegradable blends of polylactic acid (PLA) and polycaprolactone (PCL) are particularly promising for biomedical devices and soft actuators. This study develops a thermo-mechanical theoretical model to investigate the shape-memory behavior of a PLA/PCL composite blend under controlled thermal cycling. The framework integrates transient heat transfer, temperature-dependent elasticity, and viscoelastic dynamics to predict temperature evolution, deformation, and internal stress. The thermal response is computed via Newton’s law of convection, while the mechanical transition is described by a sigmoidal temperature- and crystallinity-dependent Young’s modulus. Beam bending theory is employed to evaluate the spatial distribution of strain and stress. A parametric sensitivity analysis was performed to evaluate the influence of different parameters, including the crystallinity grade, convective heat transfer coefficient, glass transition temperature, and viscoelastic recovery constant. The theoretical study accurately reproduces the shape-memory cycle, quantifying performance through fixation and recovery ratios. This model provides a robust tool for the rational design and optimization of biodegradable smart polymer structures.

## 1. Introduction

Shape memory polymers (SMPs) represent a rapidly expanding class of smart materials capable of recovering a predefined permanent shape after undergoing large, reversible deformations triggered by external stimuli such as temperature, light, moisture, or electric fields [1,2]. Their ability to store and release mechanical energy, combined with low density, large recoverable strain, tunable transition temperature, and relatively simple processing, makes SMPs attractive for applications spanning biomedical devices, aerospace deployable structures, soft robotics, and adaptive engineering systems [3,4,5]. Compared with traditional shape memory alloys, SMPs offer significantly higher deformability, lower cost, and greater design flexibility, although typically at the expense of lower recovery stress [6]. The shape memory effect in polymers is generally governed by the interplay between molecular architecture and thermally activated phase transitions. In thermally responsive SMPs, reversible switching is typically controlled by the glass transition temperature or melting transition of one or more polymer phases, which regulate chain mobility and energy storage mechanisms [7]. During programming, deformation is imposed at an elevated temperature when the material is compliant, followed by cooling to fix a temporary configuration. Subsequent reheating restores chain mobility, enabling recovery of the original shape through entropy-driven relaxation processes [8,9,10]. The efficiency of shape fixation and recovery is therefore intrinsically linked to temperature-dependent mechanical properties, viscoelastic relaxation, and phase morphology. Recent research has increasingly focused on multi-phase and multi-component SMP systems designed to enhance mechanical performance, actuation control, and functional tunability [11]. In particular, polymer blends and semi-crystalline networks have attracted substantial attention because they allow independent control of structural and switching domains through phase separation and crystallization mechanisms [12,13]. Among these, biodegradable polymer systems are of growing interest due to environmental sustainability concerns and biomedical compatibility requirements. Blends based on polylactic acid (PLA) and polycaprolactone (PCL) represent a prominent example of such materials, combining the relatively high stiffness of PLA with the ductility and low melting temperature of PCL [14,15,16,17]. Despite being immiscible, these blends can exhibit strong interfacial adhesion and cooperative thermomechanical behavior, leading to effective shape memory performance across repeated thermal cycles [18]. Experimental investigations have demonstrated that composition plays a critical role in determining thermomechanical response, stiffness evolution, and recovery capability in PLA/PCL systems. For instance, specific blend ratios can optimize the balance between structural rigidity and switching efficiency, thereby maximizing reversible deformation and shape recovery performance [16,19].

Beyond material composition, processing routes such as extrusion, hot pressing, and additive manufacturing significantly influence morphology, crystallinity, and phase distribution, further affecting shape memory behavior [20]. While experimental findings underscore the intricate coupling between microstructure and the thermomechanical response of modern SMPs, empirical characterization alone cannot fully capture the non-linear interactions between temperature-dependent elasticity, viscoelastic relaxation, and phase transitions. As SMP architectures become increasingly complex, predictive modeling tools are now essential to transcend purely empirical approaches and enable the rational design and optimization of high-performance smart materials. Over the past two decades, numerous constitutive modeling frameworks have been proposed to describe SMP behavior.

Moving from early thermomechanical models based on temperature-dependent elastic moduli, subsequent research introduced viscoelastic formulations to better capture time-dependent relaxation processes and frozen strain storage [21]. More advanced approaches have incorporated phase transition thermodynamics, internal state variables, and finite deformation kinematics to describe large strain behavior and multiphase interactions [22]. In parallel, finite element implementations of SMP constitutive laws have enabled simulation of thermomechanical cycling under realistic boundary conditions, supporting the design of smart structures and adaptive components. Recent developments include three-dimensional viscoelastic frameworks, multi-phase continuum models, and storage strain-based formulations capable of reproducing experimentally observed responses under complex loading paths [23,24,25]. Despite the advances achieved so far, a major challenge remains in bridging experimental characterization and predictive simulation in a unified, quantitatively validated framework. Many models rely heavily on curve fitting or are validated only under limited loading conditions, reducing their predictive reliability when applied to new material compositions or thermal histories.

This limitation is particularly critical for biodegradable SMP blends like PLA/PCL systems. In these materials, morphology-dependent mechanical response, thermal transitions, and time-dependent relaxation interact across multiple scales, making the numerical modeling of their shape-memory behavior mainly challenging. In particular, simplified thermo-mechanical models capable of capturing the coupled interaction between thermal cycles and heat transfer, temperature-dependent mechanical properties, and viscoelastic recovery dynamics are still needed to support the design and optimization of shape-memory structures within a single and consistent thermodynamic framework. The integration of rigorous experimental validation with computational modeling is establishing a new paradigm in the development of advanced shape memory polymers (SMPs). By bridging the gap between empirical characterization and predictive results, this synergistic framework enables systematic sensitivity analysis and virtual prototyping, significantly accelerating material optimization cycles. Consequently, the development of robust, physically grounded models for biodegradable blends is essential to decode the complex thermo-mechanical mechanisms governing shape fixation and recovery, ultimately providing a quantitative foundation for the design of next-generation smart materials.

This work presents a robust thermo-mechanical framework designed to resolve the shape-memory kinetics of PLA/PCL composite architectures. By integrating convective heat-transfer physics with temperature-dependent constitutive laws and viscoelastic relaxation dynamics, the model captures the multiscale interplay between thermal gradients and stress–strain evolution. Experimental data from our previous study serve as the foundational reference for both parameter estimation and the rigorous validation of theoretical predictions [16]. Unlike purely empirical approaches, this formulation leverages classical beam theory to map internal stress distributions and quantify performance through rigorous fixation and recovery metrics. A comprehensive sensitivity analysis was conducted both to evaluate the impact of uncertainties in the assumed parameters and to identify the most critical factors governing the recovery process, elucidating the coupling between thermal activation and macromolecular relaxation.

The resulting computational framework provides a high-fidelity tool for the predictive design and virtual prototyping of biodegradable smart structures, offering critical insights into the mechanisms that dictate structural stability and shape-switching efficiency.

## 2. Materials and Methods

### 2.1. Materials

The preparation of materials and their experimental characterization followed a previously reported procedure [16]. A concise summary of this protocol is provided below for reference. Ingeo™ 3D870-grade polylactic acid (PLA), characterized by a melt mass-flow rate of 9–15 g/10 min (210 °C/2.16 kg), was supplied by Nature Works (Plymouth, MN, USA). Polycaprolactone (PCL), with a molecular weight (M_n_) of 70,000–90,000 g/mol and an MFI of 2–4 g/10 min (160 °C/5.00 kg), was procured from Sigma Aldrich (St. Louis, MI, USA). Both polymers were utilized in pellet form. The crystallinity degrees for the pristine PLA (14.3%) and PCL (42.6%) were previously determined via DSC, as detailed in [17].

### 2.2. Preparation of PLA/PCL Composite Filaments

PLA and PCL pellets were vacuum-dried for six hours at 80 °C and 40 °C, respectively, to ensure optimal material quality. The dried polymers were then manually blended in four distinct weight ratios (95/5, 70/30, 60/40, and 30/70 PLA/PCL wt%) within sealed containers to achieve a preliminary homogeneous distribution. Melt-processing was performed using a Process 11 co-rotating twin-screw extruder (Thermo Scientific, Waltham, MA, USA). The extrusion temperature profile was maintained between 175 °C and 190 °C, with the die set at 180 °C. The screw speed was kept constant at 150 rpm, resulting in a torque range of 60–70%. Following extrusion, the composite filaments were cooled through two consecutive water baths at 65 °C and 25 °C, respectively. This controlled cooling produced solidified filaments with a nominal diameter of 1.75 ± 0.5 mm for all compositions.

### 2.3. Experimental Shape Memory Process

Rectangular strips (60 × 10 × 1 mm) were carved from thin polymer sheets produced by thermally compressing extruded filaments. The shape memory properties of the various composite blends were analyzed in bending mode utilizing a custom-designed laboratory device. Following the definition of the initial permanent state, the strain response of each thermo-responsive sample was meticulously recorded during a series of thermal fluctuations consisting of four distinct steps. In more detail, the specimens were first conditioned for 10 s in a water reservoir preheated to 60 °C, a value aligned with the thermal transitions identified via DSC—specifically, the 59.7 °C glass transition of PLA and the 63.8 °C melting point of PCL [26].

**Figure 1:**
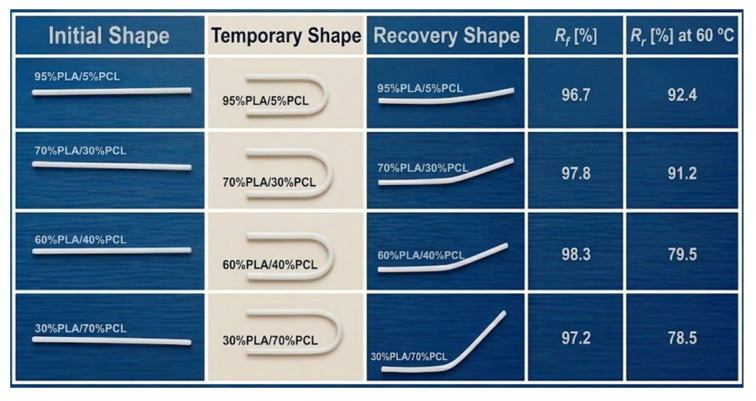
Visual representation of the shape memory of PLA/PCL blends, including the shape fixation (*R_f_*) and recovery (*R_r_*) ratios at 60 °C.

During this phase, a rigid brace was utilized to deform the submerged strip to a 180° angle. Subsequently, while preserving the applied deformation, the material was quickly transferred to a 5 °C cooling bath for a duration of 10 s. Once the mechanical constraint was withdrawn, the resulting temporary bending angle was documented as *θ_f_*. After a 20 min equilibration period at ambient temperature, the sample was reintroduced into the 60 °C bath to trigger the restoration of its permanent geometry, with the final recovered angle recorded as *θ_perm_*. Both *θ_f_* and *θ_perm_* were accurately quantified through digital image processing using ImageJ software (version 1.54k), providing the necessary data to determine the shape fixation (*R_f_*) and recovery (*R_r_*) performance according to the following analytical expressions:

(1) $$R_{f}=\frac{\theta_{f}}{\theta_{max}}\cdot 100\%,\;R_{r}=\frac{\theta_{f-}\theta_{perm}}{\theta_{max}}\cdot 100\%$$

where *R_f_* quantifies the fixation of the temporary shape, and *R_r_* quantifies recovery efficiency. In the present study, a schematic representation of these configurations, indicating the key performance parameters for the shape memory effect, is reproduced in Figure 1. To maintain focus on the model formulation, only the experimental data essential for the subsequent discussion are reported herein; full experimental details can be found in our earlier study [16].

### 2.4. Theoretical Study

In the following subsubsections, the fundamental physics and the governing constitutive equations are systematically detailed to establish a rigorous analytical framework. Starting from an adapted linear solid approach, by integrating the principles of continuum mechanics with polymer thermodynamics, the discussion progresses from the micro-scale interactions within the PLA/PCL blends to the formulation of a comprehensive theoretical model. This mathematical derivation aims to fully capture the underlying mechanisms of the shape-memory effect, accurately describing both the shape-fixing and shape-recovery stages that define the material’s functional response.

#### 2.4.1. Adapted Standard Linear Solid Model for Shape Memory Polymers: Theoretical Framework

**Figure 2:**
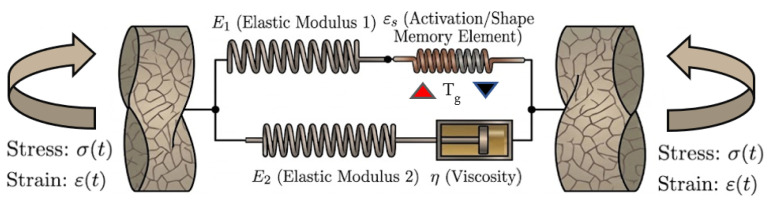
Schematic representation of the Adapted Standard Linear Solid model (ASLS) used to interpret the shape memory effect of tailored PLA/PCL blends under U-bending. Specifically, the framework integrates a shape-memory branch (*E*_1_, *ε_s_*) acting as a thermal switch for elastic strain storage, in parallel with a Maxwell viscoelastic arm (*E*_2_, *η*) governing temperature-dependent recovery kinetics. The external arrows represent the applied bending moments.

The macroscopic thermomechanical behavior of the PLA/PCL blend is described by a constitutive differential equation derived from a modified Standard Linear Solid (mSLS) model (see Figure 2). Deviating from conventional three-parameter viscoelastic frameworks [27,28], our model incorporates a thermal activation plastic-viscous strain element ($\varepsilon_{s}$) in series with the primary elastic spring (*E*_1_), according to a four-element configuration involving a slip mechanism proposed by Tobushi et al. [29]. Specifically, this element remains dormant when the temperature is below the glass transition temperature *T_g_* or the internal stress does not meet the yielding condition. Conversely, it is triggered once the temperature exceeds *T_g_* and the driving stress surpasses the temperature-dependent yield threshold (*σ_y_*).

The proposed rheological model effectively captures the thermomechanical response of the PLA/PCL blends under investigation. This architecture enables a direct representation of the ‘frozen’ strain within the PLA matrix by decoupling stored entropic energy from the dissipative mechanisms inherent to the PCL phase. Specifically, the upper branch serves as the material’s ‘elastic skeleton,’ representing the memory effect and matrix elasticity of the continuous PLA phase; it physically accounts for the storage of elastic energy and the ability to fix a temporary shape, subsequently recovered via thermal activation above *T_g_*. In parallel, the lower branch characterizes the viscoelasticity and damping properties of the PCL phase through a Maxwell element (*E*_2_ and *η*). This component captures viscoelastic chain sliding and the subsequent delayed mechanical response. Given its rubbery state at room temperature, the PCL phase enhances the blend’s dissipative capacity (*η*) and mitigates PLA brittleness, thereby governing the characteristic relaxation dynamics of the system.

The resulting analytical equation effectively captures the interplay between the macroscopic stress (*σ*) and its first-order time derivative ($\dot{\sigma }$), correlating the elastic storage branches (*E*_1_, *E*_2_) with viscous dissipation (*η*) and the temperature-dependent activation strain element (*ε_s_*), according to the following relationship:

(2) $$\sigma +\frac{\eta }{E_{2}}\dot{\sigma }=E_{1}\left(\varepsilon -\varepsilon_{s}\right)+\eta \left(1+\frac{E_{1}}{E_{2}}\right)\dot{\varepsilon }-\eta \frac{E_{1}}{E_{2}}{\dot{\varepsilon }}_{s}$$

where $\dot{\varepsilon }$ and ${\dot{\varepsilon }}_{s}$ denote the time derivatives of the total strain *ε* and activation strains, respectively. Specifically, ${\dot{\varepsilon }}_{s}$ represents the evolution rate of the internal state variable, capturing the kinetics of the strain storage and release mechanisms as the material crosses its transition temperature. See Section S1 of the Supplementary Information for details about its mathematical derivation.

To simulate the complete shape-memory cycle, this general formulation is specialized into three distinct operational phases by applying specific boundary conditions and thermal constraints:

1. Shape Programming Phase—During the initial loading at *T* > *T_g_*, a constant stress *σ*_0_ rate is imposed on the specimen. In this regime, the thermal activation plastic-viscous element *ε_s_* is progressively engaged, allowing the primary elastic branch (*E*_1_) to store the mechanical energy required for the subsequent recovery. The differential equation reduces to a linear growth form, where the material response is dominated by the simultaneous contribution of the elastic springs and the viscous dashpot, resulting in the linear increase in the deformation *ε*. See Section S1.1 of the Supplementary Information for mathematical details.
2. Cooling and Storage Phase—Once the maximum deformation *θ_max_* is reached, the external stress is removed ($\sigma =0,\;\dot{\sigma }=0$) while the temperature is rapidly decreased below *T_g_*. In this “dormant” state, a thermal activation function *α*(*T*, *χ*), which is also dependent on the degree of crystallinity *χ*, drops to zero, effectively “locking” the internal state variable (*ε_s_*) by nulling its evolution (${\dot{\varepsilon }}_{s}=0$). At these temperatures, the high internal viscosity *η* suppresses macromolecular relaxation, thereby preserving the programmed shape near its peak value, with only a marginal instantaneous elastic rebound occurring upon unloading. See Section S1.2 of the Supplementary Information for mathematical details.
3. Shape Recovery Phase—Upon reheating the specimen above the activation threshold, the internal energy barrier is overcome, and the activation element is “unlocked” (${\dot{\varepsilon }}_{s}$ ≠ 0). Under zero-stress conditions (σ=0), the energy stored in the elastic branch E_1_ during the programming phase is released, driving the strain recovery toward the permanent set $\varepsilon_{perm}$, where the effective relaxation time $\tau_{rel}=\eta /E_{2}$ dictates the recovery kinetics. See Section S1.3 of the Supplementary Information for mathematical details.

In our theoretical setup, the sample is deformed into a U-shape. Under the assumption of Euler–Bernoulli beam theory, the surface strain *ε* is proportional to the bending angle *θ*. Consequently, the macroscopic response of the material can be modeled by substituting *ε* with *θ* in Equation (2). This approach treats the angular recovery as a direct proxy for strain recovery kinetics, assuming a geometric analogy between linear and angular deformation as follows:

(3) $$\varepsilon =\frac{T_{h}}{2\cdot R}\approx \frac{T_{h}\cdot \theta }{2L}$$

where *T_h_* is the sample thickness, *R* is the radius of curvature, and *L* is the length of the deformed segment. Given that *T_h_* and *L* remain constant during the test, *ε* scales linearly with *θ* (ε∝θ).

#### 2.4.2. Sample Geometry

The specimens comply with the dimensions adopted for the experimental testing, featuring a rectangular geometry of 60 × 10 × 1 mm (*L*, *W*, and *T_h_*, respectively). The volume *V* and total surface area *A* are:

(4) $$V=L\cdot W\cdot T_{h},\;A=2\left(L\cdot W+L\cdot T_{h}+W\cdot T_{h}\right)$$

These geometric parameters are essential for the calculation of convective heat transfer with the surrounding thermal bath.

#### 2.4.3. Constitutive Material Parameters

Strategic calibration of the constitutive model relies on the integration of the intrinsic properties detailed in Table 1. Beyond the general blend morphology, these specific thermo-viscoelastic parameters—primarily the glassy/rubbery moduli and glass transition temperatures—define the mechanical threshold of the networks. Moreover, thermal properties such as density (*ρ*) and specific heat (*C_p_*) enable the model to account for the heat transfer dynamics within the composite beam. Collectively, these inputs allow the framework to accurately map molecular relaxation phenomena onto the observable shape-memory cycle of the PLA/PCL specimens. A comprehensive summary of the key parameters utilized in this study is provided in Table S1 of the Supplementary Information, which includes additional details such as nominal values, symbols, units, variability ranges for the sensitivity analysis, and whether each parameter is derived experimentally, from literature, or via model calibration.

The effective thermophysical properties of the composite—specifically density ($\rho_{eff}$), specific heat capacity ($C_{P}^{eff}$), and degree of crystallinity ($\chi_{blend}$)—were estimated via a linear rule of mixtures assuming a homogeneous phase distribution:

(5) $$P_{eff}=\omega_{PLA}\cdot P_{PLA}+\omega_{PCL}\cdot P_{PCL}$$

where *P* represents the generic property, while $\omega_{PLA}$ and $\omega_{PCL}$ represent the weight percentage of the constituent polymers. For the crystalline fraction, the model was calibrated using experimental DSC benchmarks for the neat components (*χ_PLA_* = 14.3% and *χ_PCL_* = 42.6%, as reported in [16,17]), ensuring consistency at the compositional boundaries.

These linear refinements allow the model to predict the physical properties of intermediate biphasic compositions while consistently converging to the single-phase experimental benchmarks for $\omega_{PLA}=0$ or $\omega_{PLA}=1$.

The effective glass transition temperature ($T_{g,eff}$) of the switching phase was constrained within the 38–55 °C range to account for the partial miscibility of the PLA/PCL system. This rationale ensures high shape fixity (*R_f_*); an activation threshold below this range would trigger premature chain mobility at ambient storage, leading to undesirable athermal recovery. The upper bound (55 °C) accounts for the PLA-rich phase *T_g_*, marginally depressed by the PCL plasticizing effect, while the lower bound (38 °C) represents the stability threshold required to prevent spontaneous relaxation, consistent with the *T_g_* plateau observed in partially immiscible biphasic blends.

Moreover, incorporating variable crystallinity into the thermomechanical framework accurately quantifies the reinforcing effects exerted by crystalline domains within both glassy and rubbery regimes. This approach successfully captures the evolution of Young’s modulus, thereby predicting the recovery kinetics and associated timescales of the shape-memory cycle.

Specifically, in this theoretical model, elastic moduli are modeled through a combination of the Voigt Rule of Mixtures—to capture the crystalline reinforcing effect in the glassy state—and an empirical crystallinity-based correction for the rubbery regime.

For the effective *χ*-dependent glassy modulus $E_{glassy,\;eff}\left(\chi \right)$, the semi-crystalline blend is conceptualized as a heterogeneous system consisting of a high-stiffness crystalline phase (*E_c_*) and a compliant amorphous matrix (*E_a_*). Under the assumption of parallel mechanical coupling, the effective *χ*-dependent glassy modulus $E_{glassy,\;eff}\left(\chi \right)$ is expressed as:

(6) $$E_{glassy,eff}\left(\chi \right)=\omega_{PLA}\cdot \left[E_{C,PLA}\cdot \chi_{PLA}+E_{a,PLA}\cdot \left(1-\chi_{PLA}\right)\right]+\;+\omega_{PLC}\cdot \left[E_{C,PCL}\cdot \chi_{PCL}+E_{a,PCL}\cdot \left(1-\chi_{PCL}\right)\right]$$

The coefficients $E_{c,PLA}$ and $E_{a,PLA}$ represent the crystalline and amorphous moduli of PLA, while $E_{c,PCL}$ and $E_{a,PCL}$ refer to the respective phases of PCL. The values for these parameters were sourced from experimental data reported in our earlier work [16] and are summarized as follows: $E_{c,PLA}$ = 4.31 GPa, $E_{a,PLA}$ = 266.4 MPa, $E_{C,PCL}$ = 154 MPa, and $E_{a,PCL}$ = 76.7 MPa.

Notably, the high values for the PLA crystalline phase (on the order of GPa) confirm that it serves as the primary structural component responsible for the mechanical stiffness of the hybrid system, whereas the PCL values reflect the rubbery nature of its amorphous phase at room temperature, ensuring the physical consistency between the model and the experimental setup. Given the significantly different orders of magnitude between these coefficients, the terms associated with the PCL phase could theoretically be neglected without substantial loss of accuracy. Nevertheless, these parameters are explicitly retained in our model to ensure physical completeness and to provide a comprehensive description of the mechanical contributions from both polymer components.

However, as highlighted in our previous study [16], the experimental values for these parameters are susceptible to variations depending on the test working velocity.

Consequently, a comprehensive sensitivity and uncertainty analysis was performed by perturbing their nominal values *E_c_*_,*nom*_ and *E_a_*_,*nom*_ by ±5% and ±10%. This approach was carefully conducted to investigate the model’s stability and to assess how such experimental fluctuations propagate through the predicted mechanical response.

Crucially, the rubber modulus *E_rubbery_*_,*eff*_(*χ*) was coupled to the crystallinity *χ* through a quadratic reinforcement factor in order to simulate the role of crystalline lamellae as physical cross-links in the amorphous matrix according to the equation:

(7) $$E_{rubbery,eff}\left(\chi \right)=E_{rubbery,base}\cdot \left(1+\gamma \chi^{2}\right)$$

where $E_{rubbery,base}$ represents the baseline modulus calculated according to the linear rule of mixtures, while γ (here = 10) represents the reinforcement efficiency of the crystalline phase.

The quadratic dependency and the magnitude of the reinforcement efficiency (γ = 10) are consistent with classical micromechanical frameworks for particle-reinforced elastomers and semi-crystalline polymers (such as the Guth–Gold or modified Halpin–Tsai formulations). In these theories, the higher-order structural coefficients typically fall within the range of 2 to 15, depending on the aspect ratio and structural anisotropy of the reinforcing domains—in this case, the crystalline lamellae. A value of γ = 10 sits squarely within this theoretical boundary [30,31,32]. In any case, to mitigate the impact of this assumption, a sensitivity analysis for the target property, $E_{rubbery,eff}\left(\chi \right)$, is conducted in the upcoming Section 3, evaluating scenarios where its nominal calculated value is both doubled and halved.

Despite the substantial *E_glassy_*/*E_rubbery_* ratio (*E_glassy_* ~ 10^9^ Pa and *E_rubbery_* ~ 10^7^ Pa), the *χ*-dependence of the glassy modulus is explicitly retained. While its influence is secondary compared to the orders-of-magnitude transitions in the rubbery regime, this approach ensures formal completeness and predictive accuracy across all structural transitions. A sensitivity analysis further validates the model stability and quantifies the impact of crystalline constraints on actuation dynamics.

To conclude, Young’s modulus $E\left(T,\chi \right)$ of the composite varies with temperature due to the glass-to-rubber transition of the polymer matrix and with the crystallinity grade *χ*. The corresponding mathematical function that captures the polymer’s behavior, i.e., rigid below *T_g_* and softening above *T_g_*, is described by a sigmoidal function:

(8) $$E\left(T,\chi \right)=E_{rubbery,\;eff}\left(\chi \right)+\frac{E_{glassy,\;eff\left(\chi \right)}-E_{rubbery,\;eff}\left(\chi \right)}{1+exp\left(\frac{T-T_{g,eff}}{{∆T}_{ts}}\right)}$$

where Δ*T_ts_* (here set to 1.35 °C) defines the width of the transition region, which governs the transition sharpness. The adoption of this expanded formulation is essential for the physical completeness of the model. While a simplified Young’s modulus formula provides a first approximation, it fails to account for the residual stress contributions and thermal expansion effects that are intrinsic to shape-memory polymers. Treating the stiffness as a global function *E*(*T*, *χ*) ensures a seamless mathematical transition between the glassy and rubbery regimes. This ensures that the framework remains robust and predictive across the entire operational temperature range, providing a superior fit to experimental data compared to traditional uncoupled models.

For completeness, it is worth pointing out that the aforementioned transition width parameter Δ*T_ts_* was set to 1.35 °C based on the mathematical properties of the sigmoidal formulation. In a logistic step function, the temperature span required to complete the bulk of the glass-to-rubber transition (from 10% to 90% of the modulus drop) is defined by Δ*T_span_* = 2ln(9)·Δ*T_ts_* ≃ 4.4·Δ*T_ts_*. Substituting Δ*T_ts_* = 1.35 °C yields an effective transition window of approximately 6 °C, which perfectly captures the sharp, narrow temperature range over which the experimental storage modulus of the PLA-based matrix undergoes its abrupt thermal softening. Furthermore, a sensitivity analysis is included in the results section, systematically assessing how uncertainty in this specific assumption propagates to the resulting macroscopic properties.

#### 2.4.4. Thermal Bath Profile

The thermomechanical response and the shape-memory performance of the composite beam were characterized by subjecting the specimen to a precisely regulated thermal cycle within a thermostatic bath. The theoretical protocol for the bath temperature, *T_bath_*(*t*), was structured into four functional stages designed to modulate the material’s molecular mobility. Initially, the cycle commenced with an isothermal equilibration phase at 60 °C for *t* < 10 s to ensure the polymer matrix reached a stable rubbery state above its effective glass transition temperature *T_g_*_,*eff*_. This was followed by a controlled linear cooling ramp from 60 °C to 5 °C over a 10 s interval (10≤t<20 s), facilitating the vitrification of the polymer chains and the subsequent ‘fixing’ of the temporary configuration. To ensure the complete dissipation of internal thermal gradients, a cold stabilization period was maintained at a constant 5 °C for 20≤t<30 s. Finally, the thermal recovery of the permanent shape was triggered by reheating the system to 60 °C for t≥30 s, thereby providing the necessary thermal energy to activate the entropic restorative forces within the composite structure. The temporal evolution of the bath temperature *T_bath_*(*t*) is therefore analytically defined by the following piecewise function:

(9) $$T_{bath}\left(t\right)=\;\left\{\begin{array}{c} \begin{array}{c} 60\;{}^{\circ}C,t<10\;s\\ 60-5.5\left(t-10\right)\;{}^{\circ}C,10\leq t<20\;s \end{array}\;\\ 5\;{}^{\circ}C,\;20\leq t<30\;s\\ 60\;{}^{\circ}C,\;t\geq 30\;s \end{array}\right.$$

#### 2.4.5. Temperature Evolution of the Sample

The transient temperature of the composite is modeled using Newton’s law of cooling, which describes the rate of heat exchange between the sample and the surrounding environment:

(10) $$\frac{dT}{dt}=\frac{h\cdot A}{\rho_{eff}\cdot c_{p}^{eff}\cdot V}\left(T_{bath}-T\right)$$

where *h* is the convective heat transfer coefficient (200 W/m^2^K^−1^), *T* is the instantaneous temperature of the sample, and *ρ_eff_* and *c_p_^eff^* represent the effective density and specific heat, respectively.

By discretizing the time domain with a step Δ*t_s_* and introducing the characteristic thermal response time $\tau_{rt}$, the above equation can be rewritten as:

(11) $$T_{n+1}=T_{n}+\frac{{∆t}_{s}}{\tau_{rt}}\left(T_{bath,n}-T_{n}\right),\;\tau_{rt}=\frac{\rho_{eff}\cdot c_{p}^{eff}\cdot V}{h\cdot A}$$

This formulation, where $T_{n+1}$ and $T_{n}$ denote the predicted temperature at the subsequent time step and the current instantaneous temperature, respectively, captures the thermal inertia of the sample, originating from its finite mass and heat capacity.

#### 2.4.6. Thermo-Viscoelastic Model for Shape-Memory Bending in PLA/PCL Blends

The thermo-mechanical response of the PLA/PCL composite strip during the shape-memory cycle was modeled using a thermally activated viscoelastic bending formulation. Shape-memory polymers exhibit the ability to temporarily fix a mechanically imposed deformation and subsequently recover their original configuration upon thermal activation. This behavior arises from the temperature-dependent mobility of the polymer chains and the associated variation in the elastic modulus across the glass transition region. The deformation of the strip is characterized by the evolution of the bending angle *θ*(*t*), which represents the macroscopic curvature state of the specimen. During the programming stage, the sample is heated above its effective glass transition temperature *T_g_*_,*eff*_, where the material exhibits a rubbery-like behavior with relatively low stiffness and higher molecular mobility. Under these conditions, the bending angle increases progressively until a prescribed maximum deformation *θ_max_* is reached. The imposed bending can be described as follows:

(12) $$\theta \left(t\right)=\theta_{max}\cdot \frac{t}{t_{b}},\;0\leq t<t_{b}$$

where *θ*(*t*) represents the instantaneous bending angle, *t* is time, and *t_b_* denotes the characteristic bending time required to reach the programmed deformation.

Following mechanical loading, the specimen is cooled below the effective glass transition temperature of the composite material. In this temperature regime, the mobility of polymer chains is strongly reduced, and the material transitions to a glassy state, while the effective elastic modulus increases significantly. As a consequence, the imposed deformation becomes temporarily frozen, and the bending angle should ideally remain constant during the fixation stage:

(13) $$\theta \left(t\right)=\theta_{max}\cdot \frac{t}{t_{b}},\;0\leq t<t_{b}$$

until the reheating stage begins at *t*, i.e., the instant time *t_r_*.

In reality, in contrast to ideal shape memory frameworks that assume a perfectly rigid fixation, the present model incorporates the elastic backspring and viscoelastic relaxation phenomena occurring immediately after the removal of the external constraint.

From a physical standpoint, Equation (13) is modified to describe the partial dissipation of the stored entropic energy that was not effectively ‘frozen’ during the cooling stage. This transition is governed by first-order relaxation kinetics, where the instantaneous bending angle $\theta \left(t\right)$ decays from the peak deformation $\theta_{max}$ toward a stable fixation state $\theta_{f}$ according to the following relationship:

(14) $$\theta \left(t\right)=\theta_{f}+\left(\theta_{max}-\theta_{f}\right)\cdot exp\left(-\frac{t-t_{load}}{\tau_{relax}}\right),\;t_{load}\leq t<t_{r}$$

where $\theta_{f}$ is the final tightening angle (corresponding to *θ_max_*·*R_f_*), while the parameter $t_{load}$ denotes the instant of mechanical constraint removal. From a physical perspective, at this time instant, the external stress is nullified, and the polymer chains—no longer restricted by the macroscopic constraint—begin to undergo a spontaneous rearrangement driven by the entropic elasticity of the network, driven by the parameter $\tau_{relax}$, which represents the characteristic relaxation time of the polymer network during the fixation stage. Physically, the latter quantifies the rate at which the macromolecular chains dissipate the residual elastic energy that was not effectively constrained by the glass transition.

Unlike constant-relaxation models, the present framework defines $\tau_{relax}$ as a state-dependent variable coupled to the instantaneous relative stiffness $E\left(T,\chi \right)$/$\;E_{glassy,eff}\left(\chi \right)$ for capturing the internal friction of the polymer chains on the basis of this equation:

(15) $$\tau_{relax}\left(t\right)=\tau_{0}+v_{cc}\cdot \;\frac{E\left(T,\chi \right)}{E_{glassy,eff}\left(\chi \right)}$$

where $\tau_{0}$ represents the instantaneous elastic response time (baseline offset, equal to 0.5 s in the present study), and $v_{cc}$ is a viscoelastic coupling coefficient, assumed to be 2 here. Additionally, a sensitivity analysis is presented in the results section for this property to highlight the influence of the model calibration choice and its constituent variables.

This coupling captures the physics of the ‘freezing’ process: as the temperature drops and the elastic modulus increases toward the glassy plateau *E*(*T*, *χ*) → *E_glassy_*_,*eff*_, the molecular mobility is progressively hindered by internal friction, leading to an increase in $\tau_{relax}$. From a rheological perspective, this dependency accounts for the time-temperature superposition principle within the fixation window. A higher $\tau_{relax}$ signifies a more stable temporary shape, as the material requires more time to undergo further spontaneous deformation, thereby defining the kinetics of the elastic backspring and the ultimate precision of the shape-memory effect.

Upon reheating, the recovery process is governed by a thermally activated relaxation mechanism. Unlike ideal models that assume an instantaneous transition at *T_g_*, the present framework introduces a continuous activation function *α*(*t*) to account for the progressive mobilization of polymer chains within the glass transition region. Above this threshold, the molecular segments regain mobility, and the internal elastic energy stored during deformation drives the recovery toward the original configuration. This continuous activation function is modeled via a sigmoid activation function $\alpha \left(T,\;\chi_{blend}\right)$ according to the equation:

(16) $$\alpha \left(T,\;\chi_{blend}\right)=\frac{1}{1+exp\left(-\frac{T\left(t\right)-\left(T_{g,eff}-\Delta T\left(\chi_{blend}\right)\right)}{\beta \left(\chi_{blend}\right)}\right)}$$

where $\beta \left(\chi_{blend}\right)$ is a smoothing parameter defining the temperature breadth of the transition, *T*(*t*) is the instantaneous temperature of the sample, and $\Delta T\left(\chi_{blend}\right)$ accounts for the thermal onset of the transition. This function ensures a continuous and physically consistent transition (avoiding numerical instabilities associated with ideal step functions) near the effective glass transition temperature $T_{g,eff}$. According to this, the recovery is physically “frozen” (*α* ≃ 0) in the glassy state and gradually “activated” (*α* → 1) as the material reaches the transition region.

Furthermore, in the present model, the activation parameters are formulated as functions of the total crystallinity $\left(\chi_{blend}\right)$ to capture its effect on the mobility of PLA/PCL blends. This coupling renders the shape-memory response both temperature-dependent and structurally sensitive, reflecting the role of the crystalline phase in modulating macromolecular flow. Specifically, higher crystallinity—typically associated with increased PCL content—results in a sharper and earlier activation of $\alpha \left(T,\;\chi_{blend}\right)$, thereby accelerating the relaxation kinetics.

In more detail, to account for the plasticizing effect induced by the PCL phase, the parameters $\beta \left(\chi_{blend}\right)$ and $\Delta T\left(\chi_{blend}\right)$ in Equation (16) are defined as linear functions with negative correlation to the total crystallinity of the blend:

(17) $$\beta \left(\chi_{blend}\right)=\beta_{0}\cdot \left(1-k_{\beta }\cdot \chi_{blend}\right),\;\Delta T\left(\chi_{blend}\right)={\Delta T}_{0}\cdot \left(1-k_{\Delta }\cdot \chi_{blend}\right)\;$$

where ${\Delta T}_{0}$ and $\beta_{0}$ represent the nominal activation offset and smoothing parameter, respectively, while $k_{\Delta }$ and $k_{\beta }$ are sensitivity coefficients that quantify the plasticizing efficiency of the crystalline domains on the molecular mobility.

Then, the recovery kinetics can be modeled through a suitable and modified first-order viscoelastic relaxation law, which accounts for the progressive mobilization of polymer chains and the experimental evidence of an incomplete recovery process that is governed by the competition between thermal and internal resistive forces. To accurately capture the shape recovery kinetics of PLA/PCL blends, the evolution of the instantaneous recovery angle *θ*(*t*) is described by:

(18) $$\frac{d\theta \left(t\right)}{dt}=-\alpha \left(T,\;\chi_{blend}\;\right)\frac{\theta \left(t\right)-\theta_{perm}}{\tau_{eff}\left(T,\chi \right)}$$

where *θ_perm_* = *θ_max_*(1 − *R_r_*) is the residual bending angle that accounts for the permanent set due to irreversible chain sliding after the recovery process is complete. In other words, this term accounts for the constraints imposed by the crystalline phases (particularly PCL) or molecular entanglements that prevent a full return to the original configuration.

In order to capture the thermo-mechanical coupling of the shape-memory effect, and differently from other standard models, the above effective relaxation time $\tau_{eff}\left(T,\chi \right)$ is assumed to dynamically scale with the instantaneous effective Young’s modulus $E\left(T,\chi \right)$ of the composite material, which varies significantly across the glass transition region, and the effective glassy modulus $E_{g,eff}$. Furthermore, it is also explicitly coupled to the degree of crystallinity (*χ*). The following relation is adopted:

(19) $$\tau_{eff}\left(T,\chi \right)=\left[\tau_{min}+\tau_{rc}\cdot \left(\frac{E\left(T,\chi \right)}{E_{g,eff}}\right)\right]\cdot \left(1+\lambda \chi \right)$$

where *τ_min_* is the minimum response time (internal viscosity limit), *τ_rc_* is a reference relaxation constant defining the intrinsic recovery timescale of the material. Moreover, in this formulation, *λ* is a semi-empirical constant representing the crystalline hindrance factor. The inclusion of *χ* acknowledges that crystalline domains (from both PCL and, in particular, PLA phases) act as physical cross-links that increase the internal viscosity, thereby slowing down the recovery kinetics compared to an amorphous system.

Notably, the evolution of the bending angle is directly coupled with the thermal history and the intrinsic mechanical properties of the composite. This is achieved through the temperature-dependent elastic modulus,$\;E\left(T,\chi \right)$, which incorporates the contributions of both PLA and PCL phases via a rule-of-mixtures formulation, as previously described.

Integrating physical consistency with computational efficiency, this thermo-viscoelastic model captures the complexities of thermally activated shape-memory behavior. It enables precise simulation of the programming–fixation–recovery sequence, ensuring that internal viscosity is honored to avoid unphysical artifacts across diverse PLA/PCL ratios.

In the proposed mSLS framework, the activation/shape memory element *ε_s_* operates in series with the elastic spring E_1_ within the upper branch. Its strain rate evolution ${\dot{\varepsilon }}_{s}$ is governed by a thermally activated relaxation mechanism driven by the local elastic strain stored in the branch, scaled by the continuous activation function $\alpha \left(T,\;\chi_{blend}\right)$ and the effective relaxation time $\tau_{eff}\left(T,\chi \right)$, in agreement with the following equation:

(20) $${\dot{\varepsilon }}_{s}=\alpha \left(T,\;\chi_{blend}\right)\cdot \frac{\varepsilon_{A}-\varepsilon_{1}}{\tau_{eff}\left(T,\chi \right)}$$

where $\varepsilon_{A}$ is the total strain of the upper branch, while $\varepsilon_{1}$ is the strain associated with the stress relative to the spring *E*_1_.

As expressed via Equation (16), the activation function $\alpha \left(T,\chi_{blend}\right)$ couples temperature, stress-induced configurations, and blend crystallinity $\chi_{blend}$ through the shifting of the effective glass transition temperature $\left(T_{g,eff}-\Delta T\left(\chi_{blend}\right)\right)$ and the smoothing parameter $\beta \left(\chi_{blend}\right)$.

When *T*(*t*) < *T_g_*_,*eff*_, *α* → 0, which implies ${\dot{\varepsilon }}_{s}$ → 0, mathematically ensuring that the stored strain remains securely “frozen” in the glassy state.

Otherwise, when *T*(*t*) > *T_g_*_,*eff*_, *α* → 1, activating the kinetic process where ${\dot{\varepsilon }}_{s}$ drives the system toward shape recovery.

#### 2.4.7. Kinetic Analysis

As a theoretical foundation, the shape recovery process in polymeric shape-memory materials is inherently a time-dependent viscoelastic phenomenon, driven by the entropic restoration of macromolecular chains toward their equilibrium configuration. As illustrated in the following equation, the recovery of the residual strain $\varepsilon \left(t\right)$ over time *t* can be fundamentally approximated by an exponential decay law:

(21) $$\varepsilon \left(t\right)=\varepsilon_{0}exp\left(-t/\tau_{eff}\left(T,\chi \right)\right)$$

where *ε*_0_ is the initial strain stored in the material after deformation. The physical driving force of this process is the instantaneous recovery rate, which defines the velocity of the entropic rebound. In our model, this instantaneous recovery rate (*I_rr_*), defined as the magnitude of the angular variation over time $\left|d\theta /dt\right|$ is characterized as the ratio between the current angular displacement from the equilibrium $\theta \left(t\right)-\theta_{perm}$, and the effective relaxation time $\tau_{eff}\left(T,\chi \right),$ according to the relation:

(22) $$I_{rr}\;=\left|\frac{d\theta }{dt}\right|=\frac{\theta \left(t\right)-\theta_{perm}}{\tau_{eff}\left(T,\chi \right)}$$

By introducing the thermal activation factor *α*(*T*), the rate is dynamically modulated to account for the “thermomechanical switch” behavior occurring during the glass transition, where the internal viscosity drops, and molecular mobility is restored. Specifically, the evolution of the recovery angle $\theta \left(t\right)$ is computed into a discrete-time numerical framework, and at each time step, it is updated as follows:

(23) $$\theta \left(t+∆t\right)\;=\theta \left(t\right)-\left[\alpha \left(T,\;\chi_{blend}\right)\cdot \frac{\theta \left(t\right)-\theta_{perm}}{\tau_{eff}\left(T,\chi \right)}\right]\cdot ∆t$$

To characterize the efficiency of the shape memory effect, a sensitivity analysis was performed by varying the bath temperature (*T_bath_*) within the range of 45 °C to 75 °C. This allows for the identification of the peak recovery rate $\left|{\dot{\theta }}_{max}\right|$, which represents the maximum kinetic energy of the entropic rebound.

In the investigated limited temperature range, the non-linear Arrhenius dependence of the recovery kinetics can be accurately approximated by a first-order Taylor expansion. By defining *T_onset_* as the thermal threshold where the recovery process is initiated (i.e., the x-axis intercept where $\left|{\dot{\theta }}_{max}\right|$ = 0), the kinetic response simplifies to a linear relationship, of the form:

(24) $$\left|{\dot{\theta }}_{max}\right|\;=\beta_{th}\left(T_{bath}-T_{onset}\right)$$

where $\beta_{th}$ expressed in deg·s^−1^·°C^−1^ defines the thermal kinetic sensitivity, quantifying the acceleration of the deployment rate per unit increase in bath temperature.

This linear approximation neglects higher-order terms of the exponential expansion, providing a robust fit (*R*^2^ used as a reliability metric) within a ±15 °C window around the nominal temperature. Physically, according to the transient thermal model described by Newton’s Law of Cooling (Equation (10)), this linearity suggests that, in this temperature range, the heating rate is governed by the temperature gradient $\left(T_{bath}-T_{onset}\right)$. Given the finite convective heat transfer coefficient (*h* = 200 W/m^2^K^−1^), the thermal relaxation time, $\tau_{eff}\left(T,\chi \right),$ acts as a physical filter that linearizes the response. In this condition, the peak recovery speed is dictated by the rate of energy inflow rather than the pure molecular mobility of the polymer chains. This linear dependency is of significant engineering interest, as it ensures highly predictable and controllable deployment velocities across the operational temperature range. Furthermore, the characteristic response time (Δ*t_rec_*) is quantitatively defined as the time required to reach 63.2% of the total recoverable angle (*θ*_63%_), consistent with first-order kinetic conventions.

#### 2.4.8. Stress–Strain Relationship

To provide a rigorous description of the blend’s mechanical behavior, the stress–strain relationship is modeled through a unified thermomechanical constitutive framework. This approach transcends simple linear elasticity by coupling the temperature-dependent stiffness with the internal strain states of the polymer network. The constitutive response is defined as follows to account for the interplay between mechanical, thermal, and inelastic strain components:

(25) $$\sigma \left(T,\chi \right)=E\left(T,\chi \right)\cdot \left[\varepsilon_{tot}-\varepsilon_{th}\left(T\right)-\varepsilon_{in}\left(T,\chi \right)\right]$$

In the proposed framework, the value of the internal resistive force per unit area (*σ*) is subjected to a saturation limit (about 52 MPa) to account for the elasto-plastic transition and the frozen stress state reached after the thermoforming cycle.

The global effective Young’s modulus *E*(*T*, *χ*) in this context serves as the primary subject of the sensitivity analysis to evaluate how uncertainties in phase moduli (*E_c_* and *E_a_*) propagate to the macroscopic stress. In the above equation, *ε_tot_* is the total applied strain. *ε_th_*(*T*) represents the thermal strain, calculated as *α*·(*T_test_* − *T_ref_*) with a coefficient of thermal expansion *α* = 1.2 × 10^−4^ K^−1^ at the test temperature *T_test_* = 25 °C and a temperature of reference *T_ref_* = 0 °C. The selection of these thermal boundary conditions is substantiated by specific physical and experimental requirements. *T_test_* was fixed at 25 °C to reflect standard ambient laboratory conditions, ensuring that both the mechanical characterization and the subsequent shape recovery (springback) measurements were conducted under a controlled, reproducible environment. Concurrently, *T_ref_* was established at 0 °C to provide a robust athermal baseline, representing a theoretical stress-free state. This choice follows a well-established phenomenological convention in thermomechanical modeling, allowing for the precise quantification of the thermal offset, $\varepsilon_{th}\left(T\right)$, accumulated as the material equilibrates from a reference state to the designated test temperature.

Finally, *ε_in_*(*T*, *χ*) denotes the inelastic or “frozen” strain, which accounts for the internal stress state and structural constraints imposed by the crystalline lamellae. In the present treatment, this parameter was set to a constant value of 1.5 × 10^−3^ to represent the characteristic residual strain of the semi-crystalline system, thereby allowing a focused assessment of the stiffness-related parameters.

By explicitly incorporating *ϵ_th_* and *ϵ_in_*, the model accurately captures the effective driving force for mechanical response and the characteristic shift in the stress–strain origin. These specific values were selected based on preliminary experimental observations, which showed a consistent residual offset in the stress–strain origin for the PLA/PCL blends.

To further substantiate the robustness of this expanded framework, a comprehensive sensitivity analysis was performed on *ε_th_* and *ε_in_*. This assessment quantifies how fluctuations in these constitutive parameters—arising from experimental uncertainties or material variability—influence the overall thermomechanical response and the predicted shift in the stress–strain origin. By isolating the contribution of each term, we demonstrate the stability of the unified framework across a broad range of operational conditions.

#### 2.4.9. Mechanical Modeling of Spatial Strain and Stress Distribution

To evaluate the thermomechanical robustness of the PLA/PCL blends, a localized elasto-plastic framework was developed to model the U-bending configuration (180° nominal angle), as illustrated by the experimental configuration of the previous Figure 1. Unlike simplified linear models, this approach accounts for the severe plastic deformation (*ϵ* ~ 16%) and the subsequent geometric recovery (springback) observed experimentally. The mechanical response of the analyzed 60 × 10 × 1 mm beam sample is modeled using a curvature approach at the bend apex. Under the Euler–Bernoulli assumption—where plane sections remain plane and perpendicular to the neutral axis—the curvature κ (m^−1^) is defined by the inner radius R_in_ and the half-thickness of the sample, rather than by the total sample length (*θ*/*L*), according to the equation:

(26) $$\;\kappa =\frac{1}{R_{in}+\frac{T_{h}}{2}}\;$$

To justify the choice of this framework under severe loading conditions, it is worth noting that a 180° U-bending with a 16% local strain implies a large deformation. However, for our bar geometry, the maximum local strain of 16% corresponds to an inner radius of *R_in_* = 2.625 mm. This yields a thickness-to-inner radius ratio (*T_h_*/*R_in_*) of 0.38. Although this ratio places the beam section at the threshold of moderately thick structures during peak bending, the high global slenderness ratio (*L*/*T_h_* = 60) ensures that the Euler–Bernoulli hypothesis remains a sufficiently accurate and widely accepted approximation to predict the macroscopic mechanical behavior and subsequent shape recovery [33].

For clarity, both strain and stress are herein regarded as spatiotemporal fields, formally expressed as *ε*(*t*, *y*) and *σ*(*t*, *y*), where *t* denotes time and y is the through-thickness coordinate measured from the neutral axis. The present section focuses on their spatial distribution across the beam thickness at a given stage of the thermomechanical cycle, namely *ε*(*y*) and *σ*(*y*); conversely, their time-dependent constitutive evolution at a fixed material position, *ε*(*t*) and *σ*(*t*), is derived separately in the Supplementary Information using the proposed rheological model.

Furthermore, under the assumption of uniform curvature along the beam, the solutions derived for the strain *ε*(*t*, *y*) during the loading, fixing, and unloading phases are directly mapped onto the macroscopic bending angle *θ*(*t*), preserving an identical temporal evolution.

The axial strain (*ε*) varies linearly across the beam thickness (*y*), ranging from maximum compression at the inner surface (*y* = −*T_h_*/2) to maximum tension at the outer surface (*y* = +*T_h_*/2). This distribution is governed by the following analytical equation:

(27) $$\varepsilon \left(y\right)=\kappa y$$

It is important to point out that this linear kinematic relationship is maintained at the bend apex under the Euler–Bernoulli assumption for thin beams. While the global U-shape represents a large displacement regime, the local cross-sectional integrity is preserved, allowing the strain to be modeled as a linear function of the distance from the neutral axis, even as the constitutive response enters the non-linear regime during the thermo-mechanical vitrification process.

Regarding the evaluation of shape memory performance, the macroscopically measured angular recovery *θ*(*t*) was employed as a direct indicator of the local strain recovery. This kinematic correlation is justified by the fact that the maximum local flexural strain at the bend apex is uniquely defined by the localized curvature (κ), according to the plane-section assumption. Because the transformation-induced unloading and subsequent phase transformation during recovery lead to a homogeneous reduction in the apex curvature, a direct geometric proportionality exists between the springback/recovery of the subtended angle and the relaxation of the outer-fiber strain. Consequently, tracking the macroscopic angle remains a reliable and widely adopted proxy for evaluating the strain-level recovery in highly localized U-bending configurations [34].

Given the high strain levels at the apex, the internal normal stress (*σ*) deviates from the linear generalized Hooke’s law once the yield threshold of the blend is reached. The present study adopts a perfectly plastic constitutive model to capture the toughening effect of the PCL content, which enables stable plastic flow:

(28) $$\;\sigma \left(y\right)=\left\{\begin{array}{c} E\left(T,\chi \right)\cdot \varepsilon \left(y\right),\;if\;\left|E\left(T,\chi \right)\cdot \varepsilon \left(y\right)\right|<\sigma_{y}\\ sgn\;\left(y\right)\cdot \sigma_{y},\;if\;\left|E\left(T,\chi \right)\cdot \varepsilon \left(y\right)\right|\geq \;\sigma_{y}\; \end{array}\right.\;$$

where *σ_y_* is the calibrated yield stress of the blend (*σ_y_* ~ 52 MPa). This saturation prevents unphysical stress predictions and accurately describes the formation of plastic hinges.

The bending moment (*M*), representing the internal resistive couple resulting from the stress distribution, is calculated by integrating the stress over the cross-section area:

(29) $$M=W\cdot \int_{-T_{h}/2}^{+T_{h}/2}\sigma \left(y\right)\cdot ydy$$

Upon release of the applied load, the elastic core of the sample (*σ* < $\sigma_{y}$) drives a partial geometric recovery. The springback factor *k_s_*, representing the ratio between the final fixed angle (*θ_f_*) and the initial imposed maximum angle (*θ_max_* = 180°), is determined by the residual moment capacity:

(30) $$k_{s}=\frac{\theta_{f}}{\theta_{max}}=1-\frac{M}{E\left(T,\chi \right)\cdot I\cdot \kappa }$$

where *I* = (*W*·*T_h_*^3^)/12 is the second moment of area for the rectangular cross-section. Finally, to quantify the blend’s ability to withstand severe deformation without fracture, the plastic dissipated energy density (*U_diss_*) is evaluated. The total work per unit volume is partitioned into recoverable elastic energy (*U_el_*) and dissipated plastic work:

(31) $$U_{diss}\left(y\right)=\int_{0}^{\varepsilon \left(y\right)}\sigma \left(\varepsilon^{\prime }\right)d\varepsilon^{\prime }-\frac{1}{2}\frac{\sigma {\left(y\right)}^{2}}{E\left(T,\chi \right)}$$

This energetic mapping reveals that the PCL phase acts as a high-capacity energy sink at the outer fiber, effectively shielding the sample from brittle failure during the U-shape formation.

## 3. Results

This section introduces a robust theoretical framework developed to accurately reproduce the shape memory process and its underlying thermomechanical mechanisms. The proposed model has been extensively validated against experimental data, where the consistently low error margins confirm its accuracy and strong predictive validity for real-world applications. To ensure the model’s reliability, a rigorous sensitivity analysis was systematically performed, investigating how uncertainties and variations in key material parameters affect the recovery evolution. This analytical approach provides a precise quantification of parameter influence, demonstrating the model’s stability and its ability to predict the functional response of the system across diverse transformation cycles. To offer a comprehensive visualization of these interactions, the results are presented through a combination of two-dimensional plots and complementary 3D surface maps.

### 3.1. Temperature Profiles, Modulus Evolution, and Bending Angles over Time

**Figure 3:**
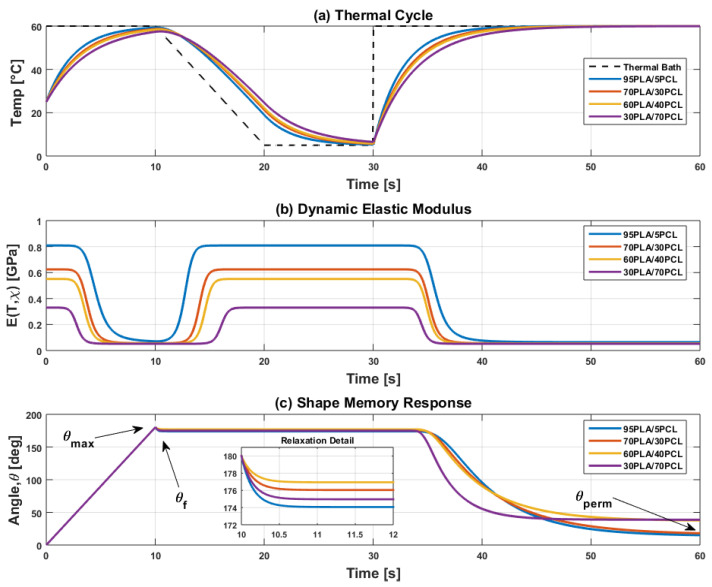
Thermomechanical simulation of PLA/PCL blends. (**a**) Thermal profiles illustrating the internal thermal lag relative to the heating/cooling bath; (**b**) Dynamic evolution of the elastic modulus (*E*) showing the shift and rubbery plateau modulation; (**c**) Shape memory response (*θ*) highlighting the composition-dependent recovery onset and kinetics. Increasing PCL content induces an earlier, more gradual recovery due to the plasticizing effect and increased viscous dissipation.

The thermomechanical performance of the PLA/PCL blends was systematically evaluated through the proposed theoretical model, as depicted in Figure 3. The multi-panel plot integrates the thermal, mechanical, and shape-memory responses to provide a comprehensive overview of the material dynamics.

Figure 3a shows the comparison between the imposed bath temperature and the simulated temperature of the composite specimens. Due to the finite heat capacity of the materials and the convective heat transfer with the surrounding environment, the specimen temperatures do not instantaneously follow the bath temperature. Instead, the temperature evolutions exhibit a characteristic delay governed by the thermal response time (*τ_rt_*) of Equation (11).

The simulation results indicate that the composite temperatures progressively approach the bath temperature with an exponential response typical of convective heat transfer systems. These delayed responses are particularly evident during the cooling stage, where the internal temperature remains higher than the bath temperature for several seconds. In particular, the numerical thermal profiles (Figure 3a) exhibit a distinct dependency on the blend composition, specifically showing a slower thermal response as the PCL content increases. This behavior can be attributed to the variation in the thermal diffusivity of the composite. As the PCL fraction increases, the higher energy required to promote molecular mobility—combined with the distinct thermal resistance of the PCL domains—induces a thermal lag relative to the external bath temperature. These results ensure that the theoretical model accurately reflects the slower heat propagation observed in PCL-rich mixtures, which is crucial for predicting the exact onset of the shape memory effect, as the material’s relaxation is strictly coupled to its instantaneous local temperature.

The variation in Young’s modulus as a function of temperature is illustrated in Figure 3b. The sigmoidal function used to describe the glass transition produces a rapid decrease in the elastic modulus near the effective glass transition temperature *T_g,eff_*. Below this temperature, the composite exhibits a glassy behavior characterized by a high elastic modulus on the order of several gigapascals. Above the transition region, the modulus decreases by approximately two orders of magnitude, reaching values typical of the rubbery state. This strong stiffness variation is the fundamental mechanism enabling the shape-memory effect. When the material is heated above *T_g_*, the polymer chains gain mobility, and the material becomes deformable. Conversely, cooling below *T_g_* freezes the polymer network, fixing the temporary shape at the final deformation angle *θ_f_*. The addition of PCL slightly lowers the effective glass transition temperature and reduces the modulus in the glassy region, although the overall mechanical response remains largely governed by PLA. The simulated bending evolution of the composite beam is presented in Figure 3c, illustrating a thermomechanical cycle composed of three distinct stages. During the loading phase (0–10 s), the beam is gradually bent from 0° to 180° while the temperature is maintained above the effective glass transition region (*T* > *T_g_*_,*eff*_); in this state, the reduced elastic modulus allows for significant deformation without large stress accumulation. In the subsequent shape fixation stage (10–30 s), the beam maintains a constant bending angle while the temperature decreases. As the material cools below the glass transition temperature, the polymer chains become effectively immobilized, locking the temporary shape into a stable configuration.

Upon reheating in the shape recovery phase, the temperature again rises above the transition threshold, triggering the release of stored elastic energy and causing the beam to progressively return toward its original configuration. This recovery follows an exponential decay characteristic of viscoelastic relaxation processes. By examining the curves, it becomes evident that the concentration of Polycaprolactone (PCL) plays a fundamental role in defining both the timing and the speed of the shape restoration, i.e., the shape recovery kinetics. An increase in the PCL fraction toward the 30PLA/70PCL composition (purple line) induces a noticeably faster recovery onset compared to the 95PLA/5PCL blend (blue line). These accelerated dynamics are primarily attributed to the plasticizing effect of PCL, which enhances molecular mobility within the PLA matrix and lowers the energy barrier for the transition from the temporary to the permanent shape. However, this increased speed is accompanied by a reduction in the overall recovery extent, as evidenced by the higher residual angles in PCL-rich blends. Such behavior suggests that while PCL facilitates faster chain relaxation, it also introduces significant viscous dissipation. The higher PCL concentrations likely promote irreversible plastic flow and internal friction, which dissipate part of the stored elastic energy and prevent the system from fully returning to its original configuration. Consequently, the blend composition acts as a critical tuning parameter to balance the competing requirements of rapid actuation and high recovery efficiency.

**Figure 4:**
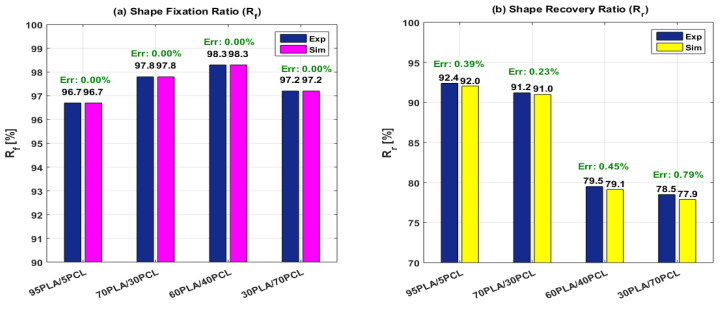
Experimental validation of the constitutive model for various PLA/PCL blends: (**a**) Shape Fixation Ratio (*R_f_*) and (**b**) Shape Recovery Ratio (*R_r_*). The comparison between experimental data (Exp) and numerical simulations (Sim) demonstrates a high predictive accuracy, with a maximum deviation of 0.79%.

The reliability of the constitutive theoretical model was validated by comparing numerical results with experimental data across all PLA/PCL blends as depicted in Figure 4. It is important to point out that the null error in R_f_, as shown in Figure 4a, is not a predictive outcome but a foundational calibration step, designed to eliminate any bias in the starting point of the recovery kinetics and to avoid propagation errors from the cooling phase, thereby allowing for a more rigorous validation of the Shape Recovery ratio (*R_r_*) and its temperature-dependent kinetics. Specifically, the experimental values of the fixed angle (*θ_f_*) were utilized as prescribed initial conditions to ensure that the elastic energy (*E*_1_) stored in the primary branch is precisely mapped to the real stored deformation of each specific blend, thus exactly defining the internal state of the shape-memory elements (*ε_s_*) before the recovery phase. The Shape Recovery Ratio (*R_r_*) in Figure 4b maintains high fidelity with a maximum deviation of only 0.79%, effectively predicting the impact of PCL content on recovery performance. This consistency suggests that the analytical definitions of the thermal activation function (*α*[*T*, *χ*]) and the effective relaxation times $\tau_{eff}\left(T,\chi \right)$ are correctly calibrated to account for the plasticizing effect of PCL and the variations in crystallinity.

The validation focused on the Shape Recovery Ratio (*R_r_*) is highly representative of the model’s predictive capability because *R_r_* is not a static property, but a dynamic macroscopic response that directly depends on the evolution of the material’s internal state and the temperature-dependent relaxation kinetics of each specific blend. Effectively predicting *R_r_* requires the constitutive model to accurately capture the complex interplay between thermal activation, phase distribution, and macromolecular mobility during the recovery phase. Ultimately, the remarkable agreement between the simulated outcomes and experimental measurements underscores the model’s reliability as a predictive tool for the advanced design of 4D-printed actuators and smart thermo-responsive devices, where precise control over the shape memory effect is mandatory.

**Figure 5:**
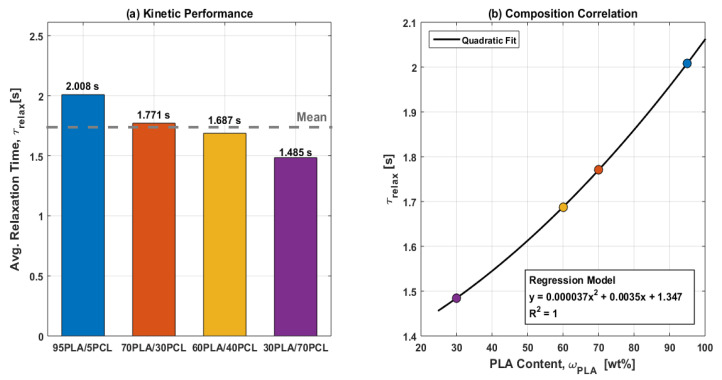
Relaxation dynamics vs. PLA content. (**a**) Comparison of fixing times across discrete blends, highlighting a 26% reduction at the 30PLA/70PCL ratio. (**b**) Parabolic correlation (*R*^2^ = 1.000) between relaxation time and *ω_PLA_*, showing the transition from a glassy-dominated barrier to a PCL-induced molecular lubrication optimum.

Furthermore, this methodological approach enables a precise quantitative evaluation of the relaxation times required to reach the experimentally observed fixed state, providing deeper insight into the material’s stabilization dynamics. Figure 5 illustrates the average relaxation kinetics during the fixing phase as a function of the PLA content. The data is presented through two complementary perspectives: Figure 5a compares the relaxation kinetics across discrete blend compositions, while Figure 5b establishes the mathematical correlation between the PLA loading and the stabilization dynamics. As shown in Figure 5a, the relaxation time exhibits a clear dependency on the blend composition. The sample with the highest PLA content (95PLA/5PCL) requires the longest time to stabilize (2.008 s), whereas the most flexible formulation (30PLA/70PCL) shows the fastest kinetics with a relaxation time of 1.485 s, representing a 26% reduction in stabilization latency. This behavior is further analyzed in Figure 5b, where the data are fitted with a quadratic model yielding a near-perfect correlation coefficient of *R*^2^ = 1. This confirms that the stabilization kinetics do not scale linearly with composition but are governed by a second-order dependency related to the evolving stiffness of the matrix.

At high PLA concentrations (95 wt%), the system exhibits the maximum relaxation time, which is primarily dictated by the glassy nature of the PLA matrix. In this regime, the high glass transition temperature (*T_g_*) and the dense entanglement network of the PLA chains impose a significant kinetic barrier, restricting the cooperative rearrangements required to “freeze” the temporary shape. As the PLA content decreases, the introduction of the PCL phase effectively acts as a high-mobility plasticizer. This increases the fractional free volume and provides a ‘molecular lubrication’ effect that facilitates faster conformational changes. However, the quadratic nature of the trend suggests that the acceleration of the stabilization process is more pronounced at intermediate PLA levels. For formulations rich in PCL (towards 30 wt% PLA), the relaxation time approaches a lower asymptotic limit dictated by the inherent viscosity of the PCL phase. Notably, the absence of a local minimum indicates that the plasticization effect of PCL is the dominant factor across the entire composition range, with no evidence of phase-separation-induced kinetic rebounding. This highlights a predictable and tunable kinetic response, where the stabilization speed can be precisely engineered by adjusting the PLA/PCL weight ratio.

Based on these preliminary insights, hereafter the theoretical analysis will focus exclusively on the 95PLA/5PCL formulation, as it demonstrated the superior performance in terms of shape recovery efficiency compared to other investigated blends. This specific composition provides the optimal balance between the structural reinforcement offered by the crystalline phase and the macromolecular mobility required for a rapid and complete recovery.

It is worth noting that the superior shape recovery efficiency of the 95PLA/5PCL blend comes at the expense of its recovery speed, highlighting an intrinsic trade-off between recovery completeness and kinetics. As illustrated by the slower recovery curve in Figure 3c, the lower PCL content restricts macromolecular mobility and extends relaxation times compared to the more flexible, PCL-rich formulations. Nevertheless, for the targeted engineering applications—such as 4D-printed actuators and precise smart devices—recovery completeness is prioritized over velocity. Maximizing the recovery ratio (*R_r_*) ensures high dimensional fidelity, precise stroke execution, and structural repeatability, which are crucial for operational accuracy, whereas faster but incomplete actuation would induce unwanted positional drift.

**Figure 6:**
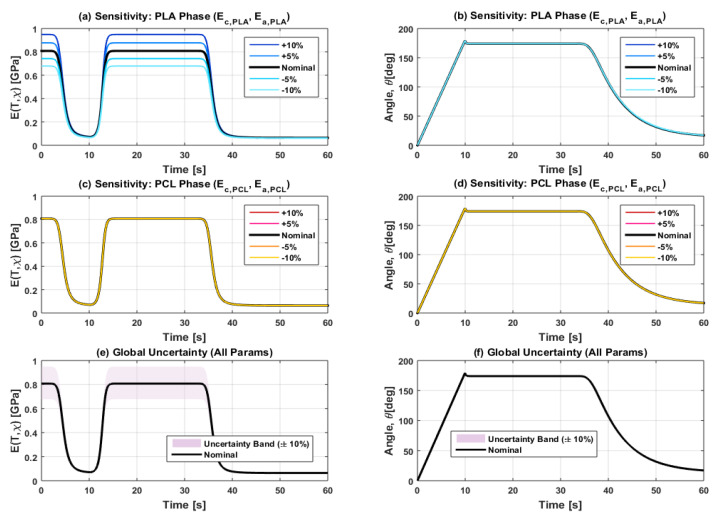
Parametric sensitivity and global uncertainty analysis of the thermomechanical model. (**a**) Evolution of the dynamic elastic modulus (*E*′) and (**b**) shape memory recovery angle (*θ*) under ±5% and ±10% fluctuations of the PLA crystalline and amorphous moduli (*E_c_*_,*PLA*_ and *E_a_*_,*PLA*_). (**c**) Sensitivity of the dynamic modulus and (**d**) angular recovery response to parametric variations in the PCL phase properties (*E_c_*_,*PCL*_ and *E_a_*_,*PCL*_). (**e**) Global uncertainty assessment for the dynamic modulus and (**f**) the corresponding shape memory response, illustrating the cumulative effect of a simultaneous ±10% variation across all input parameters; the shaded gray areas represent the uncertainty bands, while the solid black lines indicate the nominal model performance as a function of time.

Figure 6 illustrates a comprehensive sensitivity analysis of the proposed thermomechanical model, evaluating the impact of parametric variations ±5% and ±10% on the dynamic elastic modulus (*E*′) and the corresponding shape memory response (Angle, *θ*).

Regarding the PLA phase (Figure 6a,b), the results reveal that the glassy-state stiffness is highly sensitive to the crystalline and amorphous moduli (*E_c_*_,*PLA*_ and *E_a_*_,*PLA*_), with the *E*′ plateau values shifting proportionally to the input variations. However, the recovery kinetics, represented by the evolution of the angle *θ*, remain remarkably stable, suggesting that while the PLA phase dictates the absolute mechanical strength, the recovery path is intrinsically governed by the glass transition temperature, which remains unaffected by these specific stiffness fluctuations.

In contrast, the system exhibits a high degree of insensitivity to the PCL phase properties (*E_c_*_,*PCL*_ and *E_a_*_,*PCL*_), as demonstrated by the nearly overlapping curves in Figure 6c,d. The sensitivity analysis reveals that the global mechanical response in the glassy state is significantly dominated by the PLA phase. Variations in the crystalline and amorphous moduli of the PCL phase (*E_c_*_,*PCL*_ and *E_a_*_,*PCL*_) result in negligible shifts in the recovery profile, demonstrating that the shape memory effect in the 95PLA/5PCL blend is structurally robust against minor fluctuations in the secondary phase properties of PCL.

The global uncertainty analysis, depicted in Figure 6e,f, demonstrates the remarkable robustness of the thermomechanical model under a simultaneous 10% parametric variation. The narrow uncertainty band for the dynamic modulus (*E*), see Figure 6f, confirms that the glassy-to-rubbery transition is numerically stable and largely insensitive to minor fluctuations in phase-specific properties. More importantly, the angular response (Figure 6e) shows negligible variance during the programming and fixing stages, ensuring a deterministic control of the temporary shape. The slight broadening of the uncertainty area during the recovery phase reflects the cumulative sensitivity of the activation kinetics, yet the model maintains high predictive precision for the final recovery trajectory. Since the relaxation time is driven by the normalized modulus ratio, the kinetic response is invariant to global scaling of the elastic constants, ensuring consistent shape memory performance. These results highlight the framework’s reliability for the design of 4D-printed components, where material property variability is a critical factor.

### 3.2. Sensitivity of Shape Recovery Kinetics to Crystallinity: A Parametric Evaluation

**Figure 7:**
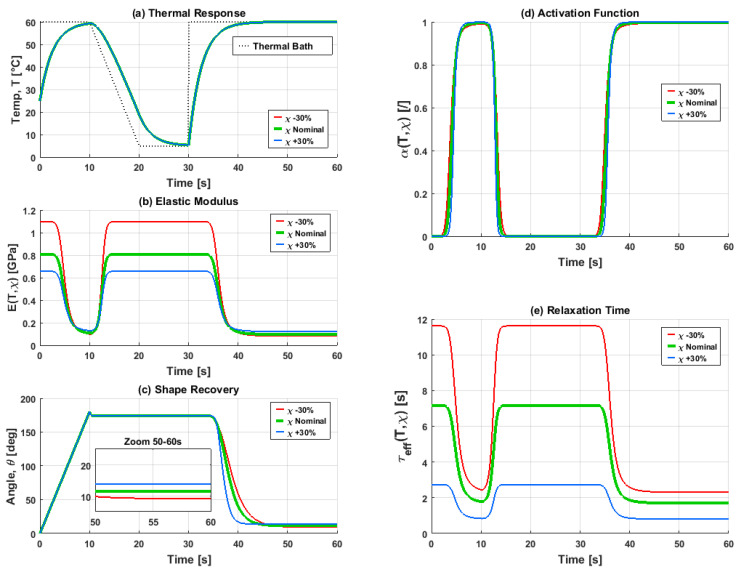
Sensitivity analysis of the 95PLA/5PCL shape-memory response. (**a**) Invariant thermal response across formulations. (**b**) Dynamic elastic modulus showing PCL-induced softening and rubbery reinforcement. (**c**) Shape recovery kinetics: higher crystallinity (blue) accelerates relaxation; inset (50–60 s) highlights the slight increase in permanent set. (**d**) Activation function *α*(*T*, *χ*) showing dominant thermal triggering with marginal *χ*-dependency. (**e**) Relaxation time quantifying the accelerated molecular mobility promoted by the crystalline content.

The degree of crystallinity (*χ*) is not merely a structural parameter but a fundamental regulator of the effective physical phenomena involved in the shape-memory polymer process. To evaluate the impact of crystalline constraints on the thermomechanical response, a parametric sensitivity analysis was conducted by varying the effective degree of crystallinity across three representative levels: a nominal value (*χ_nominal_*) and two shifted states representing a ±30% variation. This study aims to quantify how a balanced crystalline fraction modulates the trade-off between molecular mobility and recovery efficiency. The comprehensive thermomechanical response under these varying crystalline values is illustrated in Figure 7. As shown in Figure 7a, the thermal response curves for the different *χ* values are almost perfectly overlapped. This is physically consistent with the fact that the specimen temperature is primarily governed by the sample mass and the convective heat exchange with the thermal bath—parameters that remain substantially unaffected by variations in crystallinity. This overlap is strategically important, as it ensures that any observed differences in the recovery kinetics are not induced by thermal gradients but are intrinsic to the material’s microstructure. This structural influence is clearly reflected in the evolution of the elastic modulus (*E*), which captures the phase transition of the PLA/PCL blend (Figure 7b).

The model successfully captures the drastic drop in stiffness—from the GPa range to the MPa plateau—upon crossing the glass transition temperature (*T_g_* ≃ 54.15 °C). Notably, at the beginning of the cycle (*T* ≃ 25 °C), the formulation with +30% *χ* (blue curve) exhibits the lowest modulus.

This is scientifically justified by the plasticizing effect of the PCL phase, which, despite its high crystallinity, increases the free volume and chain mobility of the blend in the glassy state.

Conversely, at the end of the process (*t* = 60 s), the higher *χ* levels lead to a slightly higher rubbery plateau, confirming that crystalline domains eventually transition into reinforcing physical cross-links once the amorphous matrix is mobilized.

The impact of these structural features is most evident in the shape recovery curves shown in Figure 7c.

While all samples initiate recovery at *t* ≃ 35 s (the point at which the instantaneous temperature of the sample exceeds the activation threshold), the kinetics are significantly differentiated by *χ*. In this specific blend, higher crystallinity (blue curve) leads to a faster recovery rate. This behavior is attributed to the enhanced molecular mobility provided by the PCL chains, which act as a kinetic accelerator during the reheating phase.

However, as shown in the inset zoom of Figure 7c, the highest value also results in a slightly higher permanent set (*θ_perm_*), as the abundance of crystals may eventually hinder the complete conformational reorganization of the network, effectively “locking” a portion of the deformation (see the inset zoom in the same figure) due to the “crystalline hindrance effect”.

The mechanical triggering of this process is governed by the thermal activation function (*α*[*T*, *χ*]) shown in Figure 7d. Unlike purely thermal models, our formulation couples *α* to *χ*, showing that higher crystallinity slightly shifts the activation toward earlier timescales and marginally increases the slope of the transition. This function *α*(*T*, *χ*) acts as a binary-like switch, confirming that recovery remains dormant during the cooling and storage phases (10 s < *t* < 30 s) and becomes fully active (*α* ≃ 1) only when the thermal energy is sufficient to overcome the energy barrier of the glass transition.

Crucially, *α*(*T*, *χ*) is only slightly dependent on crystallinity, representing a purely thermal “awakening” of the polymer network when the specimen temperature crosses the *T_g_* region. Finally, the physical origin of the different recovery based on the *χ*-content is elucidated by the effective relaxation time $\tau_{eff}\left(T,\chi \right)$ in Figure 7e. The downward shift in the *τ_eff_* curves with increasing *χ* quantifies the “plasticizing drag” reduction.

By explicitly coupling *τ_eff_* and α to *χ*, the proposed model transcends empirical fitting, offering a predictive tool for tailoring the shape memory response through controlled crystallization during processing.

This sensitivity analysis proves that in 95PLA/5PCL blends, crystallinity acts as a kinetic governor, providing a robust pathway for the rational design of programmable actuators with precisely tuned response times.

**Figure 8:**
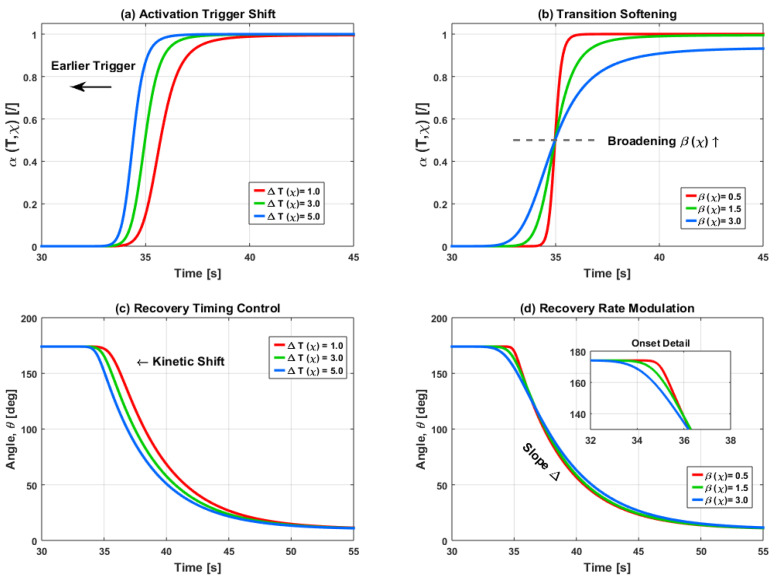
Parametric sensitivity analysis of the activation function. (**a**) Influence of the thermal onset offset (Δ*T*(*χ*)) on the trigger timing, illustrating a significant “Earlier Trigger” effect. (**b**) Influence of the smoothing parameter (*β*) on the transition breadth, demonstrating the “Broadening” of the activation profile. (**c**) Macroscopic “Kinetic Shift” in shape recovery induced by Δ*T* variations. (**d**) Recovery rate modulation via *β*(*χ*), highlighting the fine-tuning of the transition “knee” (see inset for onset detail). Red, green, and blue curves represent increasing parameter values, confirming the model’s ability to independently regulate recovery timing and kinetics.

To further elucidate the mathematical robustness and physical interpretability of the proposed model, a comprehensive sensitivity analysis was performed on the activation parameters, focusing on their decoupled influence on the shape-memory response. As illustrated in Figure 8, this study systematically isolates the roles of the thermal onset offset (Δ*T*(*χ*)) and the transition smoothing parameter (*β*(*χ*)), quantifying their individual contributions to the triggering and kinetics of the recovery process. The influence of the thermal onset offset (Δ*T*(*χ*)) on the microscopic awakening of the polymer network is clearly captured in Figure 8a. Increasing Δ*T*(*χ*) from 1.0 °C to 5.0 °C induces a substantial horizontal translation of the activation function *α*(*T*, *χ*), explicitly labeled as an “Earlier Trigger.” This temporal shift is a direct consequence of the lowered thermal energy barrier required to mobilize the amorphous matrix. Complementarily, the smoothing parameter *β* dictates the “softness” or diffusiveness of the phase transition, as explored in Figure 8b. The “Broadening” of the activation profile is evident as *β*(*χ*) increases from 0.5 to 3.0, representing a transition from a sharp, binary-like switch to a more gradual molecular mobilization.

The macroscopic manifestation of these microscopic shifts is directly reflected in the shape recovery response. In Figure 8c, the “Kinetic Shift” annotation highlights how the variation in ΔT(*χ*) advances the onset of angular decay. Crucially, this analysis demonstrates that Δ*T*(*χ*) acts as a precise temporal regulator, modulating the exact moment the specimen awakens from its dormant storage phase without significantly altering the slope of the recovery. Finally, the “Slope Δ” observed in the recovery dynamics of Figure 8d confirms that *β*(*χ*) primarily acts as a smoothness regulator for the transition onset. While the overall recovery rate is governed by the relaxation time, *β* allows for the fine-tuning of the “Recovery Rate Modulation” at the beginning of the process. It captures, as is evident from the reported zoom-inset, the initial breadth of the glass transition region, showing that a lower *β*(*χ*) (red curve) leads to a sharper, more instantaneous start, whereas a higher *β*(*χ*) (blue curve) promotes a more gradual departure from the dormant state. The synergy between these two parameters, as demonstrated in this sensitivity framework, proves that the model can be effectively tailored to describe a wide range of PLA/PCL formulations, providing a robust predictive tool for the rational design of smart polymeric structures.

### 3.3. Stress–Strain Curves: Sensitivity Analysis and Phase Contribution

**Figure 9:**
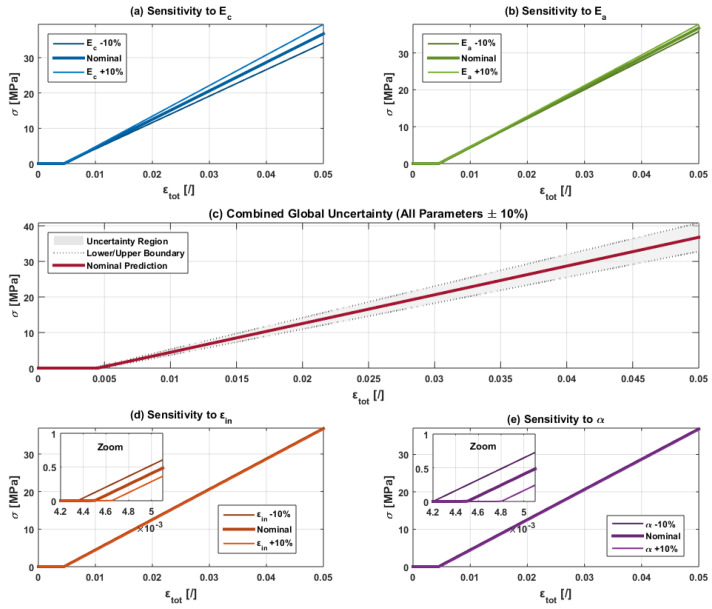
Sensitivity analysis of the blend’s mechanical response. (**a**,**b**) show the individual impact of a ±10% variation in the crystalline (*E_c_*) and amorphous (*E_a_*) phase moduli, respectively. (**c**) Displays the global uncertainty area (shaded) considering the simultaneous variation in both parameters at a calculated crystallinity of *χ* = 15.7%. Panels (**d**,**e**) quantify the sensitivity to kinematic offsets induced by the inelastic strain (*ϵ_in_*) and the thermal expansion coefficient (*α*), highlighting the model’s high robustness against horizontal shifts in the constitutive origin and its stability during the thermoforming vitrification process.

To evaluate the robustness of the proposed model and account for the inherent variability of experimentally derived elastic moduli for PLA phases, a comprehensive sensitivity analysis was performed. Accordingly, Figure 9 illustrates the mechanical response of the blend under a ±10% variation in the crystalline (*E_c_*) and amorphous (*E_a_*) moduli, both individually and combined in panels (a), (b) and (c), respectively.

The individual phase analysis reveals that the model is significantly more sensitive to the crystalline modulus, as shown in the first panel (a). Although the degree of crystallinity is lower than the amorphous fraction (*χ* ≃ 15.7%), the high intrinsic stiffness of the crystalline domains—typically reported around 4.3 GPa—means that even small percentage uncertainties in *E_c_* propagate more aggressively into the final mechanical prediction. This indicates that the crystalline phase acts as the dominant reinforcing factor, making the overall stiffness of the PLA-rich blend highly dependent on the stability of its crystalline regions. Conversely, as depicted in panel (b), the amorphous phase exhibits a much more moderate influence on the overall stress–strain slope. Despite constituting approximately 84% of the blend’s volume, its lower absolute modulus (*E_a_* ≃ 266 MPa) results in a limited shift in the effective stiffness for the same percentage of tolerance. This suggests that the mechanical response is relatively robust against fluctuations in the amorphous phase properties, provided the crystalline content remains constant. The global sensitivity analysis presented in panel (c) represents the simultaneous variation in both parameters, providing a confidence interval for the predicted mechanical response. The resulting shaded uncertainty area encapsulates the potential experimental scattering that might arise from morphological variations in the blend. The proximity of the nominal prediction (red line) to the center of this interval suggests that the model is inherently stable, though it emphasizes that precise characterization of the crystalline fraction is more critical than the amorphous phase properties for the accurate mechanical design of high-PLA content materials.

Crucially, panels (d,e) highlight a remarkable robustness of the unified framework against fluctuations in kinematic parameters. Unlike the moduli, the inelastic strain (*ϵ_in_*) and the thermal expansion coefficient (*α*) do not alter the slope but govern the horizontal translation of the constitutive origin. The visual overlap of the curves under a ±10% variation demonstrates that the proposed model is exceptionally stable, and this relative insensitivity ensures that the mechanical predictions remain reliable even in the presence of minor experimental uncertainties in the thermal or inelastic characterization of the blend. These “shift” parameters dictate the “stress-free threshold” of the material—representing the internal state captured during the thermoforming vitrification process—without compromising the overall predictive accuracy. Moreover, this negligible sensitivity to minor kinematic uncertainties ensures that the observed shape fixity and the minimal springback are governed by a reliable mechanical origin, defining the precise point at which the polymer network begins to bear macroscopic load after cooling.

To conclude, it is worth noting that, as illustrated in all sensitivity plots, the stress response remains null at low strain levels, reflecting a physical activation threshold. This plateau represents the regime where the total applied strain (*ε_tot_*) is consumed to compensate for the cumulative offsets induced by thermal expansion (*ε_th_*) and the inelastic component (*ε_in_*). Tensile loading initiates only once *ε_tot_* exceeds this internal strain state, thus highlighting the model’s ability to accurately capture the onset of mechanical response in pre-strained or thermally expanded systems.

### 3.4. Spatial Strain and Stress Distribution: Sensitivity Analysis and Phase Contribution Illustrate Internal Gradients

**Figure 10:**
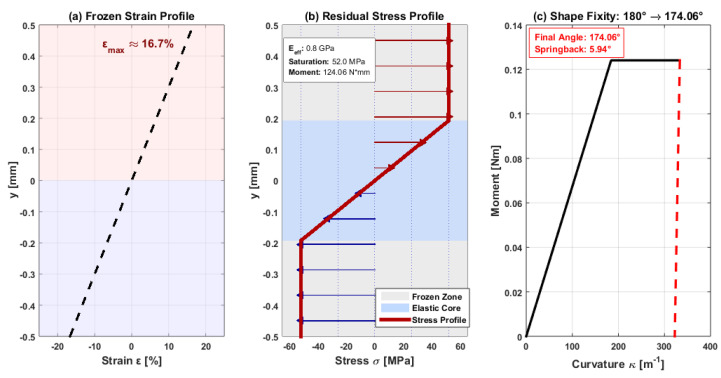
Spatial mechanical analysis of the specimen under flexural loading: (**a**) Axial strain distribution (*ϵ*) across the thickness (*ϵ_max_* ≃ 16.7%), showing a linear profile consistent with Euler–Bernoulli beam theory. (**b**) Normal stress distribution (*σ*) highlighting the Frozen Zone and the Elastic Core (blue shaded area); the red line shows the stress saturation at ±52 MPa. (**c**) Moment–curvature (*M*-*κ*) relationship: the dashed line quantifies the elastic springback (5.94°), resulting in a final fixed angle of 174.06°.

The mechanical integrity and the exceptional shape memory performance of the 95PLA/5PCL blend are quantified through the localized multi-scale analysis presented in Figure 10. Providing a spatial mapping of the internal mechanical state is scientifically imperative, as global geometric observations alone cannot elucidate the complex internal stress redistribution that governs the stability of the thermoformed U-shape. This figure serves as a fundamental bridge between the micro-scale kinematic assumptions and the macro-scale structural reliability, offering a precise visualization of how the vitrification process “locks” the macromolecular orientation induced above the glass transition temperature (*T_g_*).

In Panel (a), the frozen strain profile illustrates the linear kinematic distribution across the 1 mm specimen thickness. Despite the extreme macroscopic rotation required to achieve a 180° U-shape, the local strain field adheres to the Euler–Bernoulli assumption, where plane sections remain plane at the bend apex. The calculation reveals a significant maximum strain of approximately 16.7% at the outer fibers. The mapping clearly distinguishes between the tensile (red-shaded) and compressive (blue-shaded) regions, substantiating that the sample undergoes severe local deformation that would lead to catastrophic failure in neat, brittle PLA, but is here accommodated by the toughening effect of the 5% PCL phase. The resulting internal state after constrained cooling is detailed in the Residual Stress Profile (Panel (b)). This distribution exhibits a characteristic S-shaped trajectory, providing definitive evidence of stress saturation during the vitrification process. While the central Elastic Core (blue-shaded region) maintains a linear response, the outer Frozen Zones saturate at a residual stress limit of approximately 52 MPa. This saturation represents the maximum entropic stress that the glassy matrix can sustain at room temperature. The explicit force vectors (red and blue arrows) visualize the internal resistive couple, where the saturation plateaus act as “mechanical anchors” that stabilize the deformed state. The calibrated internal moment of 124.06 N·mm serves as the quantitative link to the global recovery behavior.

The transition from local stress to global geometric stability is finalized in the Shape Fixity Analysis (Panel (c)). The moment–curvature relationship highlights the loading path toward the localized plastic-like hinge and the subsequent elastic unloading (dashed red line) upon release. The steepness of this unloading path is a direct manifestation of the high Shape Fixity of the blend. Mathematically, the model predicts an experimental final angle of 174.06°, corresponding to a minimal springback of only 5.94°. This high fidelity between the intended and achieved geometry confirms that the majority of the mechanical work is successfully dissipated and fixed within the polymer network, with only a negligible elastic fraction remaining to drive the recovery. These results collectively demonstrate that the 95PLA/5PCL blend is an optimized candidate for complex thermoforming applications, ensuring predictable and robust geometric retention under severe curvature.

**Figure 11:**
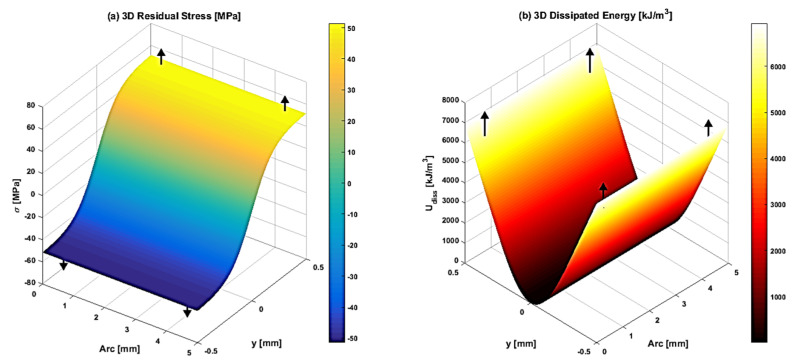
Three-dimensional thermomechanical mapping of the 95PLA/5PCL bend apex: (**a**) residual stress distribution [MPa] showing S-shaped yield saturation. The black arrows represent the directionality of the residual stress vectors frozen within the polymer network: the outward-pointing arrows at *y* = 0.5 mm denote the sustained entropic tensile state, while the inward-pointing arrows at *y* = −0.5 mm highlight the compressive regime required for curvature maintenance. (**b**) Plastic energy dissipation [kJ/m^3^] exhibiting a V-shaped profile. The narrow elastic core at the neutral axis (*y* = 0) explains the minimal 5.94° experimental springback and high shape fixity. Complementarily, the arrows emphasize the energy dissipation peaks located at the specimen’s outer sides. These vectors visually confirm that the thermoforming work is non-uniformly distributed, being maximum at the surfaces where the plastic-like deformation is most intense.

The localized thermomechanical state at the apex of the U-bent 95PLA/5PCL specimen is comprehensively illustrated in Figure 11, providing a three-dimensional spatial mapping of the internal stress field and energy dissipation patterns following the thermoforming cycle. The specimen, initially deformed at a temperature exceeding the glass transition (*T_g_*) to exploit the increased macromolecular chain mobility, was subsequently cooled under constraint to “lock” the induced geometric configuration. The localized thermomechanical state at the apex of the U-bent 95PLA/5PCL specimen is comprehensively illustrated in Figure 11, providing a three-dimensional spatial mapping of the internal stress field and energy dissipation patterns following the thermoforming cycle. The specimen, initially deformed at a temperature exceeding the glass transition (*T_g_*) to exploit the increased macromolecular chain mobility, was subsequently cooled under constraint to “lock” the induced geometric configuration. In this context, Panel (a) presents the residual normal stress distribution across the thickness, characterized by a distinct S-shaped profile that reflects the stress state captured during the vitrification process. Despite the rubbery state facilitated by the heating phase, the cooling below *T_g_* freezes a significant portion of the internal stresses, which saturate at approximately ±52 MPa at the outer fibers. This saturation confirms the formation of a stable mechanical framework where the imposed strain is balanced by the semi-crystalline network of the PLA matrix and the ductile PCL phase, preventing the unphysical stress peaks associated with purely elastic models and ensuring the structural integrity of the localized “plastic hinge.”

The physical mechanism underlying the high fidelity of the thermoformed shape is further elucidated by the volumetric energy mapping in Panel (b), which displays the V-shaped trajectory of the plastic dissipated energy density (*U_diss_*). This energy landscape highlights a profound contrast between the neutral axis (*y* = 0), characterized by a narrow “elastic-core” where dissipation is negligible, and the external surfaces, where the dissipated energy peaks at approximately 8000 kJ/m^3^. This massive energy dissipation represents the mechanical work consumed by irreversible molecular reorientation and entropic relaxation, which is effectively “fixed” during the cooling stage. The stability of the final U-shape, evidenced by an experimental springback of only 5.94° (from 180° to 174.06°), is a direct consequence of this energy-sink mechanism. The PCL fraction plays a pivotal role in this process, acting as a toughening agent that facilitates stable energy dissipation across the outer fibers, thereby shielding the specimen from brittle failure. Consequently, the limited geometric recovery is driven solely by the minimal elastic energy retained in the central filament, demonstrating the exceptional shape fixity and predictive reliability of the 95/5 blend under severe thermoforming conditions.

### 3.5. Viscoelastic Recovery, Evolution and Speed

**Figure 12:**
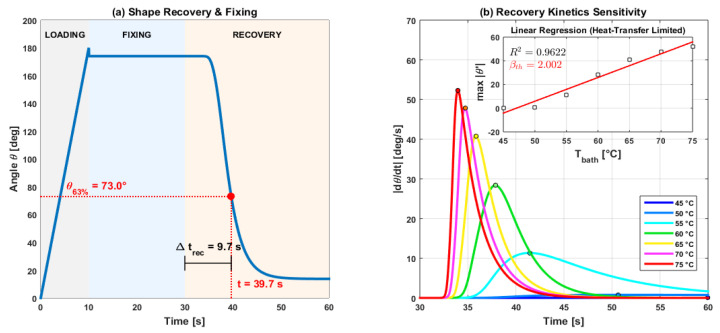
Kinetic sensitivity analysis of the shape memory actuator. (**a**) Representative thermo-mechanical recovery cycle at *T_bath_* = 60 °C. (**b**) Influence of bath temperature on the angular recovery rate. The inset shows the linear relationship (*R*^2^ = 0.9622) between the peak recovery rate and *T_bath_*, indicating a convection-limited regime. The thermal sensitivity coefficient *β_th_* = 2.002 deg·s^−1^·°C^−1^ quantifies the deterministic response of the device.

The temporal evolution of the shape memory response, aligned with the programmed thermo-mechanical cycle, is illustrated in Figure 12. The analysis highlights the transition from the shape-fixing stage to the active recovery phase, providing a comprehensive view of the material’s sensitivity to thermal triggers.

As shown in Figure 12a, by initiating the analysis at *t* = 10 s, the transition from the shape-fixing phase (10–30 s) to the active recovery phase (30–60 s) is clearly visible. During the fixing stage, the specimen maintains a stable deformation; however, a slight recovery is observed as the internal temperature equilibrates, ultimately reaching the stable fixed angle *θ_f_*. This subtle adjustment signifies the final stabilization of the polymer chains in their temporary configuration as the relaxation time effectively ‘freezes’ the entropic state. Upon the application of the 60 °C thermal trigger at *t* = 30s, a distinct latency period is observed due to the thermal lag effect, with the recovery onset occurring only after the internal temperature approaches the *T_g_* value. The characteristic decay time, measured from the trigger onset to the 63.2% recovery point (red marker), is quantified at *t* = 39.7 s (Δ*T_rec_* = 9.7 s). This value represents the synergistic effect of the specimen’s thermal inertia and the internal viscoelastic relaxation time [$\tau_{eff}\left(T,\chi \right)$].

The recovery kinetics, illustrated in Figure 12b, quantify this process through the evolution of the transformation rate. As shown by the resulting bell-shaped kinetic curves, the response highlights the competition between the thermal inertia of the specimen and the exponential acceleration of molecular relaxation. Higher bath temperatures drastically reduce the internal viscosity, shifting the peak rate to earlier time intervals and resulting in a more impulsive and complete restoration of the original geometry.

Conversely, at temperatures near the lower bound of the transition region, the recovery is dominated by high resistive forces, leading to a broader and attenuated kinetic response. The sharp peak in the transformation rate confirms that the most significant entropic release occurs within a narrow window after activation, where the maximum transformation velocity is achieved shortly after crossing the effective glass transition temperature $T_{g,eff}$.

The thermal sensitivity coefficient, denoted as *β_th_*, was numerically quantified as 2.002 deg·s^−1^·°C^−1^. This parameter serves as a macroscopic indicator of the coupling between the convective heat flux (*h*) and the structural relaxation of the SMP. Physically, *β_th_* represents the efficiency of the energy transfer. A higher value indicates a system where the thermal resistance (proportional to 1/*h*·*A*) is low compared to the mechanical stiffness of the polymer. Interestingly, the peak recovery rate exhibits a high linear correlation (*R*^2^ = 0.9622) with the bath temperature. While molecular relaxation is intrinsically non-linear (often following Arrhenius or VFT dynamics), this linearity is justified by a first-order Taylor expansion of the rate equation within the investigated temperature range. In this convection-limited regime, the kinetic response is primarily governed by the rate of energy inflow (Newton’s Law of Cooling), ensuring a highly predictable and deterministic actuation behavior.

### 3.6. Animations Depict Dynamic Shape Recovery

**Figure 13:**
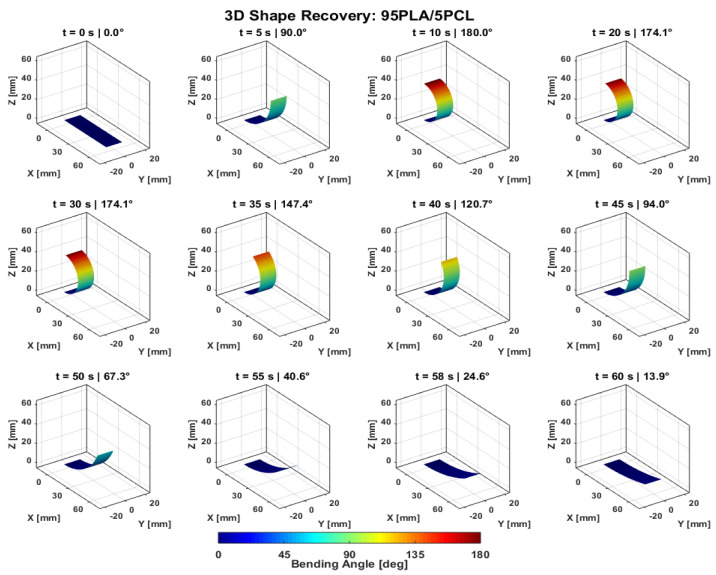
Spatiotemporal 3D evolution and actuation kinetics of the 95PLA/5PCL shape memory composite beam highlighting the transition between the various thermomechanical states.

To better illustrate the complex mechanical behavior and the intrinsic actuation kinetics of the 95PLA/5PCL composite beam, a high-resolution dynamic visualization of the bending and recovery process was generated through three-dimensional thermomechanical simulations (Figure 13). This 3D evolution provides a holistic perspective on the shape memory cycle, meticulously highlighting the transition between the various thermomechanical states.

During the initial loading stage (*t* = 0–10 s), the animations capture the gradual bending of the composite as it is programmed at *T_g_*, reaching a maximum deflection of 180°, U-turn configuration, which collapses to about 174.1° for relaxation phenomena previously discussed. This phase emphasizes the material’s high-temperature ductility and its ability to undergo severe plastic-like deformation without structural compromise. Subsequently, the visualization effectively depicts the frozen configuration maintained during the cooling and storage phase (*t* = 10–30 s). In this stage, the sudden increase in the elastic modulus ‘locks’ the polymer chains in their temporarily strained state, demonstrating an excellent shape fixity ratio with negligible elastic springback. Finally, the sequence highlights the progressive return to the original shape during the reheating phase (*t* = 30–60 s). The dynamic transition from the fully folded 174.1° configuration to a final residual angle of only 13.9° serves as a visual proof of the high recovery efficiency of the 95PLA/5PCL blend. By mapping the color gradient to the instantaneous bending angle, the simulation provides a clear spatial representation of the thermal-to-mechanical energy conversion, offering a predictive tool for the design of soft actuators where precise angular control and rapid recovery kinetics are paramount for functional reliability.

### 3.7. Sensitivity Overview of Key Parameters

**Figure 14:**
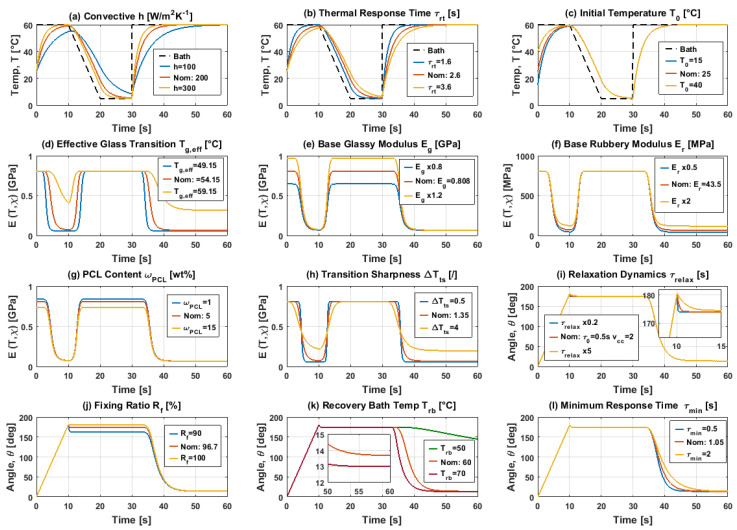
Sensitivity Overview of Key Parameters on Shape Memory Response and Mechanical Behavior of PLA/PCL Composite. The dashboard quantifies individual and synergistic effects of environmental and intrinsic parameters on SMP’s performance: (**a**) Convective h: Influence of heat transfer coefficients on the thermal equilibration speed. (**b**) Thermal Inertia or thermal response time *τ_rt_*: Impact of the material’s time constant on temperature response latency. (**c**) Initial Temperature *T*_0_: Sensitivity of the thermal cycle to the starting sample conditions (15–40 °C). (**d**) Glass Transition *T_g_*: Modulus window shifts under variations in transition temperature. (**e**) Glassy Modulus *E_g_*: Scaling effects of the glassy state stiffness on the overall mechanical cycle. (**f**) Base Rubbery *E_r_*: Sensitivity of the high-temperature and final equilibrium plateau to variations in the rubbery modulus. (**g**) PCL Content *ω_PCL_*: Plasticizing and reinforcing effects (1–15%) on the effective Young’s Modulus. (**h**) Transition Sharpness Δ*T_ts_*: Impact of the switching “sharpness” on the softening rates and final modulus baseline. (**i**) Relaxation Dynamics *τ_relax_*: Influence of viscoelastic relaxation constants on the stabilization and overshoot of the temporary shape. (**j**) Fixing Ratio *R_f_*: Model capability in tracking the maintenance of the temporary deformation across different targets. (**k**) Recovery Bath Temp T_rb_: Threshold analysis of the thermal stimulus on recovery kinetics and completeness. (**l**) Minimum Response Time *τ_min_*: Sensitivity of the recovery velocity to the internal viscoelastic resistance, defining the “promptness” of the angular return to the permanent shape.

The sensitivity of the 95PLA/5PCL composite to environmental and intrinsic variables is systematically evaluated in the master dashboard of Figure 14. The figure is organized into twelve subplots, each representing a different aspect of the material response as influenced by variations in a specific parameter. All curves show three conditions: lower bound, nominal value, and upper bound of the parameter under investigation. The results highlight that small changes in material composition or environmental conditions can have significant effects on the performance of PLA/PCL shape memory composites. This master dashboard effectively summarizes the relative influence of each parameter, providing a clear guide for material design and optimization for shape memory applications. The thermal response is primarily dictated by the convective coefficient *h* (Subplot (a)) and the thermal response time *τ_rt_* (Subplot (b)), where higher *h* values and reduced *τ_rt_* accelerate the approach to the bath temperature, minimizing the characteristic thermal lag. Subplot (c) demonstrates that variations in the initial temperature *T*_0_ introduce a temporal shift in the activation onset, although the system eventually converges toward the stimulus temperature once the activation threshold is crossed. The mechanical and structural integrity of the “frozen” state depends on the interplay between the glass transition temperature and the modulus magnitude. The thermal switching window is highly sensitive to the *T_g_* (Subplot (d)), which shifts the modulus transition along the time axis, and to the nominal glassy modulus *E_g_* (Subplot (e)), scaling the overall stiffness. Furthermore, Subplot (f) identifies the base rubbery modulus *E_r_*, which acts as a scaling factor for the material’s stiffness in its high-temperature state; specifically, it dictates the minimum modulus reached during the transition and determines the final plateau value of *E* after the shape recovery is complete (*t* > 40 s). Material composition plays a synergistic role; as evidenced in Subplot (g), increasing the PCL fraction acts as a plasticizing agent that primarily scales down the glassy modulus peaks, reducing the overall stiffness of the matrix.

The “sharpness” of this activation is controlled by the transition width Δ*T_ts_* (Subplot (h)), where lower values facilitate near-binary switching, while higher values significantly broaden the transition and lead to a higher residual modulus at equilibrium.

A pivotal observation is found in the relaxation dynamics of Subplot (i). The high-magnification inset reveals that after reaching the peak deformation, a subtle angular adjustment occurs as the polymer chains equilibrate to reach the stable fixed angle *θ_f_*. This highlights the importance of the viscoelastic relaxation time *τ_relax_* in ensuring dimensional stability. The robustness of the shape-fixing phase is validated in Subplot (j), where the model effectively tracks the material’s ability to lock in temporary deformations across different *R_f_* targets. However, the most significant performance leap is captured in Subplot (k), which illustrates the sensitivity to the Recovery Bath Temperature (*T_rb_*). The results demonstrate that increasing the thermal stimulus to 70 °C not only accelerates the kinetic response but also enhances the overall recovery efficiency. The inset detail at the 60 s mark reveals a crucial physical insight: the higher thermal energy at 70 °C enables the polymer chains to overcome internal viscous constraints more effectively, resulting in a lower residual angle compared to the nominal case, as experimentally observed in our previous study [16]. This confirms that the recovery ‘completeness’ is a temperature-driven process, where superheating serves as a catalyst for more exhaustive entropic restoration. Finally, the internal damping of the entropic release is summarized in Subplot (l), where the minimum response time (*τ_min_*) acts as the ultimate kinetic bottleneck. Even under optimal thermal inflow, a high internal friction constant forces a creeping recovery, confirming that the maximum transformation velocity is achieved only when the external thermal stimulus is matched by low internal viscoelastic resistance.

Based on this sensitivity analysis, three primary drivers emerge as dictating the functional performance of the 95PLA/5PCL actuator. These parameters represent the fundamental “tuning knobs” for tailoring the deployment speed and reliability: The Thermal Gateway: Glass Transition Temperature (*T_g_*), The Kinetic Bottleneck: Convective Coefficient (h), and The Internal Brake: Minimum Response Time (*τ_min_*). The *T_g_* is the most critical material-dependent parameter. A minor shift does not merely alter the activation timing but fundamentally changes the switching efficiency. Lowering *T_g_* (e.g., through increased PCL content) broadens the operational window but risks premature activation, while a higher *T_g_* ensures a more robust “frozen” state at the cost of a higher thermal trigger threshold. The analysis confirms that the system operates in a convection-limited regime. The convective coefficient *h* serves as the external “clock” of the actuator: regardless of the internal polymer chemistry, the recovery speed cannot exceed the rate of thermal inflow. Increasing *h* from 100 to 300 W/m^2^K^−1^ linearizes the response and drastically reduces the latency period, making it the primary engineering variable for fast-response soft robotics. While h controls the energy input, *τ_minc_* governs the internal entropic release. This viscoelastic relaxation constant acts as an internal damping mechanism. Optimizing the polymer’s molecular weight and cross-linking density to minimize *τ_min_* is therefore essential for achieving high-impulse actuation.

## 4. Conclusions

This study presents a thermo-mechanical numerical model for the analysis of the shape-memory behavior of PLA/PCL composite beams subjected to controlled thermal cycles. The proposed approach combines transient heat transfer analysis with temperature-dependent mechanical properties and viscoelastic recovery modeling in order to simulate the key stages of the shape-memory process. The theoretical results demonstrate that the temperature evolution of the specimen is strongly influenced by convective heat transfer and the thermal inertia of the composite material. The delayed response of the internal temperature relative to the external thermal bath plays a significant role in determining the timing of the mechanical transitions during the thermomechanical cycle. The temperature-dependent Young’s modulus successfully captures the transition between the glassy and rubbery states of the polymer system, which is responsible for the activation of the shape-memory effect. During heating above the effective glass transition temperature, the composite becomes compliant and allows large deformations. Subsequent cooling freezes the polymer network and fixes the temporary shape. Upon reheating, the stored elastic energy drives the recovery toward the original configuration. The spatial analysis of strain and stress distributions confirms that bending deformation generates linear stress gradients across the beam thickness, with maximum tensile and compressive stresses located at the outer surfaces. These stress concentrations are relevant for evaluating potential mechanical failure or fatigue during repeated actuation cycles. Furthermore, the viscoelastic recovery model provides a realistic description of the time-dependent evolution of residual strain during the recovery stage. The characteristic recovery time plays a key role in determining the speed of the shape-memory response. Although the present model is intentionally simplified, it captures the fundamental physical mechanisms governing thermally activated shape-memory polymers. Future work may extend this approach by incorporating non-linear viscoelastic constitutive laws, phase-dependent material parameters, and finite-element simulations of more complex geometries. Overall, the developed numerical framework provides a useful tool for understanding the thermo-mechanical behavior of biodegradable PLA/PCL composites and may assist in the design of advanced smart polymer systems for biomedical devices, soft robotics, and adaptive structures.

## Figures and Tables

**Table 1:** Additional physical properties for PLA and PCL.

Property	PLA	PCL
Density *ρ* [kg/m^3^]	1250	1150
Specific heat *C_p_* [J/kg·K]	1800	2000
Glassy modulus *E_glassy_* [Pa]	3 × 10^9^	0.4 × 10^9^
Rubbery modulus *E_rubbery_* [Pa]	4.5 × 10^7^	1.5 × 10^7^
Glass transition temperature *T_g_* [°C]	60	−60

## Data Availability

The original contributions presented in this study are included in the article/Supplementary Material. Further inquiries can be directed to the corresponding author.

## Supplementary Materials

The following supporting information can be downloaded at: https://www.mdpi.com/article/10.3390/polym18131577/s1, Figure S1: Qualitative trend of the strain evolution during the shape programming phase. The total strain response (ε(t), solid red line) exhibits a transient viscoelastic regime at t < 2 s, followed by a steady-state linear viscoplastic drift. The analytical model (dashed curve) shows excellent agreement with the rigorous numerical solution (red curve). More in detail, the dashed blue line (ε_interp_) represents the asymptotic linear interpolation, isolating the purely viscoplastic contribution (β/α)t and the residual elastic offset (−β/α^2^+δ/α) from the transient exponential recovery; Figure S2. Evolution of the variable ε(t) during the Shape Fixation Phase (t > t_1_ s). The solid magenta line represents the analytical model, asymptotically approaching the steady-state value β_1_/α (dashed black line). Figure S3. Analytical modeling of the thermally-induced strain recovery phase (T > T_g_). The blue solid line represents the temporal evolution of the total strain $\varepsilon \left(t\right)$, starting from the programmed state ε_1_ = 2 at t = 30 s. The recovery behavior is characterized by an exponential viscoelastic decay toward a permanent set ε_∞_ (dashed black line), which quantifies the residual viscoplastic deformation. The inset table provides the calibrated constitutive parameters (E_1_, E_2_, $\gamma ,\;\sigma_{y}$) and the resulting relaxation time τ = 16.0 s, showing the balance between elastic driving forces and viscoplastic dissipation. Table S1: Parameter Table.
