# Supplementary material for: Shape Memory Response of Tailored Polylactic Acid/Polycaprolactone Blends: A Validated Constitutive Theoretical Investigation and Sensitivity Analysis
Source: Polymers (Basel). 2026 Jun 25;18(13):1577. doi: 10.3390/polym18131577 (PMC13363994; doi:10.3390/polym18131577)
Supplement: Supplementary file 1 [file polymers-18-01577-s001.zip › polymers-4373305-supplementary.pdf]

polymers

MDPI

Supplementary Information

Shape Memory Response of Tailored Polylactic Acid/Polycaprolactone Blends: A Validated Constitutive Theoretical Investigation and Sensitivity Analysis

Giovanni Spinelli [1,2,*], Rosella Guarini [2,3], Evgeni Ivanov [2,4], Rumiana Kotsilkova [2] and Vittorio Romano [3]

[1] Faculty of Transport Sciences and Technologies, University of Study "Giustino Fortunato", Via Raffaele Delcogliano 12, 82100 Benevento, Italy
[2] Open Laboratory on Experimental Micro and Nano Mechanics, Institute of Mechanics, Bulgarian Academy of Sciences, Acad. G. Bonchev Str., Block 4, 1113 Sofia, Bulgaria; rguarini@imbm.bas.bg (R.G.); ivanov_evgeni@imbm.bas.bg (E.I.); kotsilkova@imbm.bas.bg (R.K.)
[3] Department of Industrial Engineering, University of Salerno, Via Giovanni Paolo II, 84084 Fisciano, Italy, vittorioromano2022@gmail.com
[4] Center of Competence for Mechatronics and Clean Technologies "Mechatronics, Innovation, Robotics, Automation and Clean Technologies"—MIRACle, 1113 Sofia, Bulgaria
* Correspondence: g.spinelli1@unifortunato.eu

S1 Shape Recovery Model with Derivation

As introduced in the main manuscript, strain and stress are formally treated as spatiotemporal fields, $\varepsilon(t,y)$ and $\sigma(t,y)$. In the present study, the through-thickness coordinate is fixed $(y=y^*)$, so that the constitutive analysis is performed using the reduced notations $\varepsilon(t,y^*)\equiv\varepsilon(t)$ and $\sigma(t,y^*)\equiv\sigma(t)$.

Furthermore, under the assumption of uniform curvature along the beam, the solutions derived for the strain $\varepsilon(t,y)$ during the loading, fixing, and unloading phases can be directly mapped onto the macroscopic bending angle $\theta(t)$. Consequently, the spatial dependence is resolved, preserving an identical temporal evolution.

In particular:

$$\boldsymbol{\varepsilon(t,y^*)=\varepsilon(t)=\kappa(t)\cdot(Th/2)} \quad \text{(S1)}$$

and

$$\boldsymbol{\theta(t)=\int_0^L \kappa(t)\ ds} \quad \text{(S2)}$$

which, in the presence of constant curvature, reduces to:

$$\theta(t)=\kappa(t)\cdot L=\left(\frac{L}{Th/2}\right)\varepsilon(t) \quad \text{(S3)}$$

The time-dependent thermomechanical shape-memory behavior of the PLA/PCL polymer blend under controlled thermal cycling was modeled using a dual-branch elasto-plastic/elasto-viscous rheological system, schematically shown in Fig. 2 of the main paper, consisting of two parallel branches designed to capture strain programming, shape fixation, and thermally activated recovery.

The upper branch, A, consists of a linear spring (E_1) in series with a thermally activated plasto-viscous strain element (ε_s), hereinafter referred to as 'slip'. The lower branch B consists of a linear spring (E_2) in series with a dashpot element (η). Specifically, in our case, this element remains dormant when the temperature is below the glass transition temperature T_g or the internal stress does not meet the yielding condition. Conversely, it is triggered once the temperature exceeds T_g and the driving stress surpasses the temperature-dependent yield threshold (σ_y).

Since branches A and B are mechanically connected in parallel between the same end nodes, kinematic compatibility requires both branches to undergo the same strain. Simultaneously, static equilibrium dictates that the externally applied stress is balanced by the sum of the internal stresses carried by each branch. Mathematically, this is expressed as:

$$\boldsymbol{\sigma} = \boldsymbol{\sigma_A} + \boldsymbol{\sigma_B} \qquad \boldsymbol{\varepsilon} = \boldsymbol{\varepsilon_A} = \boldsymbol{\varepsilon_B} \tag{S4}$$

where $\boldsymbol{\varepsilon_A}$ and $\boldsymbol{\sigma_A}$ are the elastic deformation and stress of branch A, while $\boldsymbol{\varepsilon_B}$ and $\boldsymbol{\sigma_B}$ are the plastic deformation and stress of branch B, respectively.

Considering the constitutive behavior of the constituent elements, the following relation holds for elastic springs 1 and 2:

$$\boldsymbol{\sigma_i = E_i \varepsilon_i \quad i = 1, 2} \tag{S5}$$

Where

σ_i denotes the internal stress carried by the i-th elastic spring;

E_i is the elastic modulus of the i-th spring;

$\boldsymbol{\varepsilon_i}$ is the corresponding strain, with i=1,2.

For the dashpot element (η), the following equation is valid:

$$\boldsymbol{\sigma_\eta = \eta \dot{\varepsilon}_\eta} \tag{S6}$$

Where

σ_η denotes the internal stress;

η is the viscosity;

$\dot{\boldsymbol{\varepsilon}}_{\boldsymbol{\eta}}$ is the corresponding strain rate.

In particular, for the slip element ε_s results:

$$\dot{\boldsymbol{\varepsilon}}_s = \begin{cases} \mathbf{0} & \text{if} \quad |\,\boldsymbol{\sigma}\,| < \boldsymbol{\sigma_y} \quad \textbf{and} \quad \boldsymbol{T \le T_g} \quad \textbf{locked plastic flow} \\ \boldsymbol{\gamma(\sigma_0 - \sigma_y)} & \text{if} \quad |\,\boldsymbol{\sigma}\,| > \boldsymbol{\sigma_y} \quad \textbf{and} \quad \boldsymbol{T > T_g} \quad \textbf{irreversible strain} \end{cases} \tag{S7}$$

where $\dot{\boldsymbol{\varepsilon}}_{\mathbf{s}}$ is the strain rate, and $\boldsymbol{\gamma}$ denotes a plastic mobility parameter.

For branch A (elasto-plastic), since the spring and the slip element are arranged in series, the total strain is the sum of the individual deformations, while the stresses remain equal:

$$\boldsymbol{\varepsilon_A} = \boldsymbol{\varepsilon_1} + \boldsymbol{\varepsilon_s} \qquad \boldsymbol{\sigma_A} = \boldsymbol{\sigma_1} = \boldsymbol{\sigma_s} \tag{S8}$$

where $\boldsymbol{\varepsilon_1}$ and $\boldsymbol{\sigma_1}$ are the elastic deformation and stress of the spring, respectively, whereas $\boldsymbol{\varepsilon_s}$ and $\boldsymbol{\sigma_s}$ represent the plastic strain and stress of the slip element.

Considering the constitutive equations of the individual elements:

$$\sigma_A = \sigma_1 = E_1\varepsilon_1 = E_1(\varepsilon_A - \varepsilon_s) = E_1(\varepsilon - \varepsilon_s) \tag{S9}$$

Similarly, for the visco-elastic branch B, the spring E_2 is connected in series with the viscous damper (η). Therefore, the strains are additive, and the stresses satisfy:

$$\boldsymbol{\varepsilon_B} = \boldsymbol{\varepsilon_2} + \boldsymbol{\varepsilon_\eta} \qquad \boldsymbol{\sigma_B} = \boldsymbol{\sigma_2} = \boldsymbol{\sigma_\eta} \tag{S10}$$

Differentiating with respect to time and substituting the constitutive equations yields:

$$\dot{\boldsymbol{\varepsilon}}_{\mathbf{B}} = \dot{\boldsymbol{\varepsilon}}_{\mathbf{2}} + \dot{\boldsymbol{\varepsilon}}_{\boldsymbol{\eta}} = \frac{\dot{\boldsymbol{\sigma}}_{\mathbf{2}}}{\mathbf{E_2}} + \frac{\boldsymbol{\sigma_\eta}}{\boldsymbol{\eta}} = \frac{\dot{\boldsymbol{\sigma}}_{\mathbf{B}}}{\mathbf{E_2}} + \frac{\boldsymbol{\sigma_B}}{\boldsymbol{\eta}} \tag{S11}$$

from which the following equation is derived:

$$\boldsymbol{\sigma_B} = \boldsymbol{\eta}(\dot{\boldsymbol{\varepsilon}}_{\mathbf{2}} + \dot{\boldsymbol{\varepsilon}}_{\boldsymbol{\eta}}) - \boldsymbol{\eta}\frac{\dot{\boldsymbol{\sigma}}_{\mathbf{B}}}{\mathbf{E_2}} = \boldsymbol{\eta}\dot{\boldsymbol{\varepsilon}} - \frac{\boldsymbol{\eta}}{\mathbf{E_2}}[\dot{\boldsymbol{\sigma}} - \mathbf{E_1}(\dot{\boldsymbol{\varepsilon}} - \dot{\boldsymbol{\varepsilon}}_{\mathbf{s}})] \tag{S12}$$

Finally, grouping the variable and their first-order derivatives, it is possible to obtain the final equation:

$$\boldsymbol{\sigma} + \frac{\boldsymbol{\eta}}{\mathbf{E_2}}\dot{\boldsymbol{\sigma}} = \mathbf{E_1}(\boldsymbol{\varepsilon} - \boldsymbol{\varepsilon_s}) + \boldsymbol{\eta}\left(\mathbf{1} + \frac{\mathbf{E_1}}{\mathbf{E_2}}\right)\dot{\boldsymbol{\varepsilon}} - \boldsymbol{\eta}\frac{\mathbf{E_1}}{\mathbf{E_2}}\dot{\boldsymbol{\varepsilon}}_{\mathbf{s}} \tag{S13}$$

This governing differential equation constitutes the mathematical model of the proposed dual-branch elasto-plastic/elasto-viscous rheological system for PLA/PCL blends,

simultaneously accounting for elastic energy storage, irreversible plastic, and viscous dissipation during strain programming, shape fixation, and thermally activated recovery.

S1.1 Analytical Framework for the Shape Programming Phase

To describe the material response under high-stress conditions ($\sigma = \sigma_0 > \sigma_y$), the governing equation for T>T_g is derived from the standard viscoelastic equation (S13). Under constant loading ($\sigma = \sigma_0$, hence $\dot{\sigma} = 0$), and incorporating the viscoplastic evolution laws:

$$\dot{\boldsymbol{\varepsilon}}_{\boldsymbol{s}}(\boldsymbol{t}) = \boldsymbol{\gamma}(\boldsymbol{\sigma_0} - \boldsymbol{\sigma_y}) \qquad \boldsymbol{\varepsilon}_{\boldsymbol{s}}(\boldsymbol{t}) = \boldsymbol{\gamma}(\boldsymbol{\sigma_0} - \boldsymbol{\sigma_y})\boldsymbol{t} \tag{S14}$$

The expression simplifies into a first-order non-homogeneous differential equation:

$$\frac{d\varepsilon}{dt} + \alpha\varepsilon = +\beta t + \delta \tag{S15}$$

The lumped parameters are defined as follows:

$$\alpha = \frac{E_1}{\eta\left(1 + \frac{E_1}{E_2}\right)} = \frac{1}{\tau} \qquad \beta = \frac{E_1\gamma(\sigma_0 - \sigma_y)}{\frac{E_1}{\eta\left(1 + \frac{E_1}{E_2}\right)}} \qquad \delta = \frac{\sigma_0 + \eta\frac{E_1}{E_2}\gamma(\sigma_0 - \sigma_y)}{\frac{E_1}{\eta\left(1 + \frac{E_1}{E_2}\right)}} \tag{S16}$$

where τ is the characteristic relaxation time. Integrating from $t = 0$ (at which $\varepsilon = \varepsilon_0$) to $t = t_1$ (at which $\varepsilon = \varepsilon_1$), the analytical solution for the total strain evolution is:

$$\varepsilon(t) = \left(\varepsilon_0 + \frac{\beta}{\alpha^2} - \frac{\delta}{\alpha}\right)e^{-\alpha t} + \frac{\beta}{\alpha}t + \left(-\frac{\beta}{\alpha^2} + \frac{\delta}{\alpha}\right) \quad \text{for} \quad \begin{matrix} 0 \le t \le t_1 \\ \varepsilon_0 \le \varepsilon \le \varepsilon_1 \end{matrix} \tag{S17}$$

This expression reveals that the strain response is governed by an initial exponential viscoelastic transition followed by a linear viscoplastic drift proportional to the stress over-threshold, as depicted in Figure S1, by assuming, only for example, the following parameter values: ε_0=0, α=0.5, β=2, and δ=5.

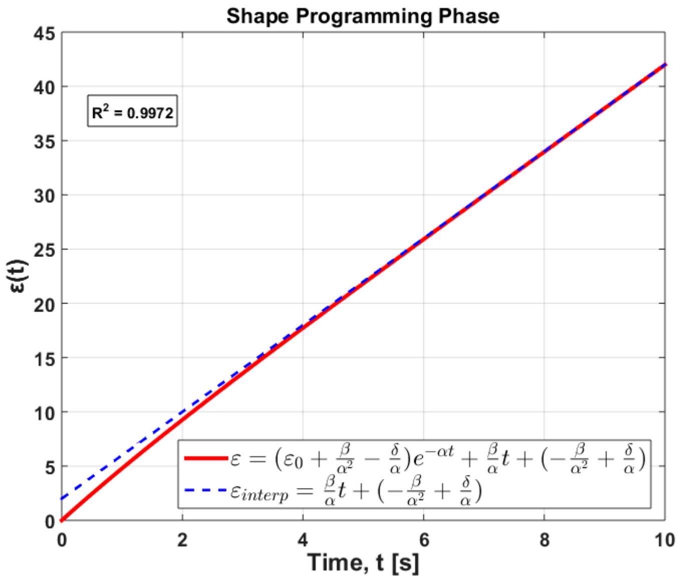

Figure S1. Qualitative trend of the strain evolution during the shape programming phase. The total strain response (ε(t), solid red line) exhibits a transient viscoelastic regime at t<2s, followed by a steady-state linear viscoplastic drift. The analytical model (dashed curve) shows excellent agreement with the rigorous numerical solution (red curve). In more detail, the dashed blue line (ε_{interp})

represents the asymptotic linear interpolation, isolating the purely viscoplastic contribution (β/α)t and the residual elastic offset (-β/α²+δ/α) from the transient exponential recovery.

S1.2 Analytical Derivation of the Shape Fixation Phase

Upon removal of the external stimulus (σ=0 and $\dot{\sigma} = 0$) at t=t_1 with T<T_g, the material enters the fixation phase $(\dot{\varepsilon}_s = 0 \; \forall \, T < T_g)$. In this regime, the viscoplastic strain becomes constant, defined as:

$$\boldsymbol{\varepsilon_{s1} = \gamma(\sigma_0 - \sigma_y)t_1} \tag{S18}$$

The governing constitutive equation (S13) simplifies to a homogeneous differential form:

$$\eta\left(1 + \frac{E_1}{E_2}\right)\frac{d\varepsilon}{dt} + E_1\varepsilon = E_1\varepsilon_s \tag{S19}$$

By introducing the previously defined relaxation parameter α and the forcing term $\beta_1 = \alpha \cdot \varepsilon_{s1}$, the equation is rewritten as:

$$\frac{d\varepsilon}{dt} + \alpha\varepsilon = \alpha\varepsilon_{s1} = \beta_1 \tag{S20}$$

The analytical solution is obtained by integrating the expression using the integrating factor $e^{\alpha t}$ over the interval [t_1, t], with the initial condition $\varepsilon(t_1) = \varepsilon_1$. The resulting time-dependent strain evolution for $t_1 \le t \le t_2$ is given by:

$$\boldsymbol{\varepsilon(t) = e^{-\alpha(t-t_1)}\left[\varepsilon_1 - \frac{\beta_1}{\alpha}\right] + \frac{\beta_1}{\alpha}} \tag{S21}$$

The governing Eq. S13 reduces to a first-order relaxation law, whose analytical solution shows that the total strain exponentially decays from its initial value ε_1 toward the residual plastic strain ε_{s1}. Physically, this behavior represents the progressive release of recoverable elastic energy through viscous dissipation. Meanwhile, the irreversible strain stored in the slip element remains preserved, stabilizing the temporary programmed shape. As time t increases, the total strain asymptotically converges toward the residual value $\varepsilon_{eq} = \varepsilon_{s1}$, which represents the temporarily fixed strain and directly quantifies the shape-fixity performance of the PLA/PCL blend, as visualized as an example in Figure S2 assuming the following generic values: t_1=10 s, ε_1=42.13, α=0.5, β_1=1, and δ=5.

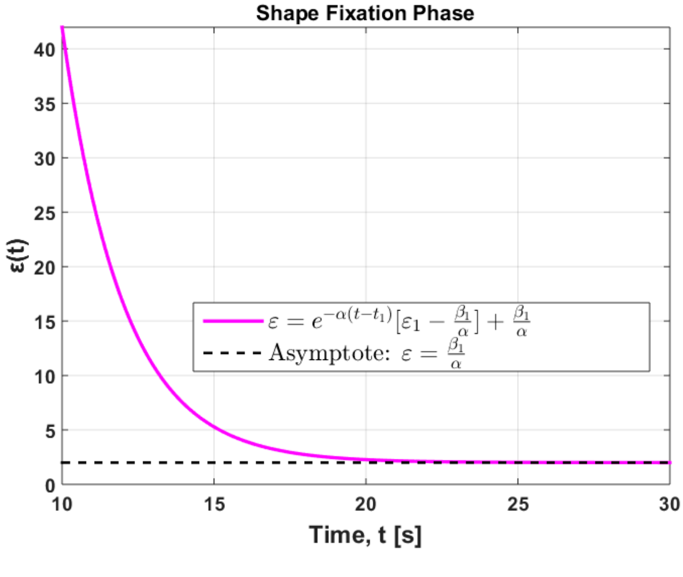

Figure S2. Evolution of the variable ε(t) during the Shape Fixation Phase ($t>t_1$ s). The solid magenta line represents the analytical model, asymptotically approaching the steady-state value β_1/α (dashed black line).

S1.3 Analytical Derivation of the Shape Recovery Kinetic

Following the Shape Fixation Phase, for $t > t_2$, the specimen is reheated above its glass-transition temperature ($T>T_g$) with zero external stress ($\sigma = 0$ and $\dot{\sigma} = 0$). Under these conditions, although the macroscopic applied stress vanishes, the internal stresses carried by the two parallel branches, A and B, remain active and satisfy the mechanical equilibrium condition ($\sigma = \sigma_A + \sigma_B = 0$, which implies $\sigma_A = -\sigma_B$).

Above the glass transition temperature $\left(T > T_g\right)$, the enhanced segmental mobility of the polymer chains re-enables slip activation; however, plastic sliding can effectively develop only as long as the residual stress in the elasto-plastic branch satisfies the yielding condition $\sigma_A > \sigma_y$. Consequently, the thermally activated recovery stage must be described through a piecewise formulation. In the first interval, $t_2 \leq t < t_y$, slip remains active while the residual internal stress progressively decreases until the critical condition $\sigma_A = \sigma_y$ is reached. In the second interval $t_y \leq t < t_{final}$, although $T > T_g$ is maintained, the residual stress falls below the yielding threshold, the slip element becomes mechanically locked ($\dot{\varepsilon}_s = 0$), and the subsequent strain evolution follows the same first-order relaxation equation previously derived for the shape-fixation stage, with updated initial conditions at t_y.

Applying the internal stress equilibrium condition ($\sigma = \sigma_A + \sigma_B = 0$, whereby $\sigma_A = -\sigma_B$) to the differential Eq. (S13) yields the governing equation for the first recovery interval:

$$\eta\left(1 + \frac{E_1}{E_2}\right)\dot{\varepsilon} + E_1(\varepsilon - \varepsilon_s) = \eta\frac{E_1}{E_2}\dot{\varepsilon}_s \tag{S22}$$

and simplifying with α:

$$\frac{d\varepsilon}{dt} + \alpha(\varepsilon - \varepsilon_s) = \frac{\eta}{E_2}\alpha\dot{\varepsilon}_s \tag{S23}$$

To solve for the total strain ε(t), we incorporate the viscoplastic flow rule $\dot{\varepsilon}_s$ according to the following relationship:

$$\boldsymbol{\dot{\varepsilon}_s = \gamma\left[E_1(\varepsilon - \varepsilon_s) - \sigma_y\right] = \gamma\left(E_1 x - \sigma_y\right)} \tag{S24}$$

and introducing the auxiliary variable:

$$\boldsymbol{x(t) = \varepsilon(t) - \varepsilon_s(t)} \tag{S25}$$

representing the effective elastic driving strain, which substituting into the constitutive relation, simplifies the system to:

$$\dot{x} + \mathrm{k}x = \mathrm{m} \ \text{ with } \begin{cases} \mathrm{k} = \left[\alpha - \left(\frac{\eta}{E_2}\alpha - 1\right)\gamma E_1\right] \\ \mathrm{m} = -\left(\frac{\eta}{E_2}\alpha - 1\right)\gamma\sigma_y \end{cases} \tag{S26}$$

Integrating via the factor e^{kt} from the initial state at the start of heating (t_2, x_2), we obtain the temporal evolution of the elastic component:

$$\boldsymbol{x(t) = \left(x_2 - \frac{m}{k}\right)e^{-k(t-t_2)} + \frac{m}{k}} \tag{S27}$$

To obtain the total strain $\varepsilon(t) = x(t) + \varepsilon_s(t)$ we calculate $\varepsilon_s(t)$ from $\dot{\varepsilon}_s(t)$:

$$\frac{d}{dt}\varepsilon_s = \gamma\left(E_1 x - \sigma_y\right) = \gamma\left\{E_1\left[\left(x_2 - \frac{m}{k}\right)e^{-k(t-t_2)} + \frac{m}{k}\right] - \sigma_y\right\} \quad \text{(S28)}$$

Integrating Eq. S28 yields:

$$\varepsilon_s = \varepsilon_s(t_2) - \gamma E_1\left(\frac{x_2}{k} - \frac{m}{k^2}\right)e^{-k(t-t_2)} + \gamma\left(E_1\frac{m}{k} - \sigma_y\right)(t - t_2) + \gamma E_1\left(\frac{x_2}{k} - \frac{m}{k^2}\right) \quad \text{(S29)}$$

Finally, we can calculate $\varepsilon(t) = x(t) + \varepsilon_s(t)$:

$$\begin{aligned}\varepsilon(t) = &\left[\left(x_2 - \frac{m}{k}\right)e^{-k(t-t_2)} + \frac{m}{k}\right] \\ &+ \left[\varepsilon_s(t_2) - \gamma E_1\left(\frac{x_2}{k} - \frac{m}{k^2}\right)e^{-k(t-t_2)} + \gamma\left(E_1\frac{m}{k} - \sigma_y\right)(t - t_2) \right. \\ &\left. + \gamma E_1\left(\frac{x_2}{k} - \frac{m}{k^2}\right)\right]\end{aligned} \quad \text{(S30)}$$

By collecting the common factors, we obtain the following solution:

$$\boldsymbol{\varepsilon(t) = Ae^{-k(t-t_2)} + B(t - t_2) + C \quad \text{for } t_2 \leq t \leq t_y} \quad \text{(S31)}$$

where the coefficients A, B, and C are functions of the material constants (E_1, E_2, γ, σ_y) and the state variables at $t=t_2$, according to the following equations:

$$A = \left[\left(x_2 - \frac{m}{k}\right) - \gamma E_1\left(\frac{x_2}{k} - \frac{m}{k^2}\right)\right] = \left(1 - \frac{\gamma E_1}{k}\right)\left(x_2 - \frac{m}{k}\right) \quad \text{(S32)}$$

$$B = \gamma\left(E_1\frac{m}{k} - \sigma_y\right) \qquad C = \left[\varepsilon_s(t_2) + \frac{m}{k} + \gamma E_1\left(\frac{x_2}{k} - \frac{m}{k^2}\right)\right] \quad \text{(S33)}$$

This analytical framework describes a complex recovery mechanism: an exponential decay representing the rapid elastic retraction, superimposed on a residual linear drift (B) and a permanent offset (C), which together quantify the incomplete recovery due to viscoplastic dissipation above T_g, according to a qualitative trend reported only as an example in Figure S3 by assuming the arbitrary values reported in the annotation box.

Finally, if and when $\sigma_A < \sigma_y$ the deformation profile evolves according to:

$$\boldsymbol{\varepsilon(t) = \varepsilon_{sy} + \left(\varepsilon_y - \varepsilon_{sy}\right)e^{-\alpha(t-t_y)} \quad \text{for } t_y \leq t \leq t_{final}} \quad \text{(S34)}$$

similar to Eq. S21, starting from the new condition ε_y at t_y

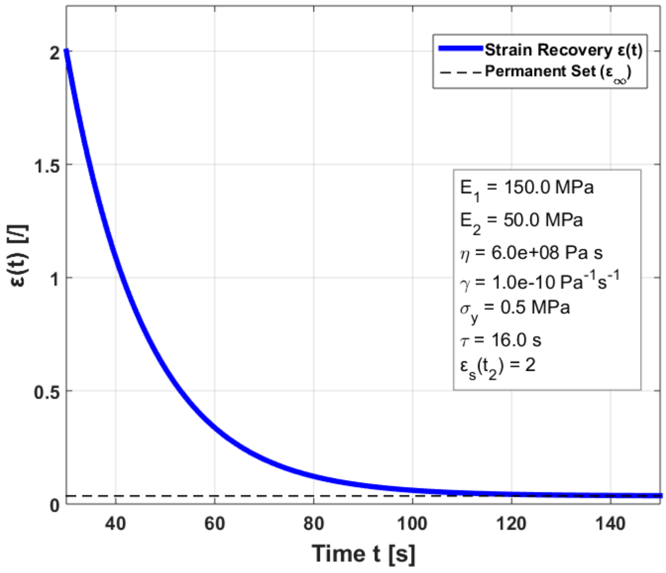

Figure S3. Analytical modeling of the thermally induced strain recovery phase ($T>T_g$). The blue solid line represents the temporal evolution of the total strain $\varepsilon(t)$, starting from the programmed state $\varepsilon_1=2$ at t=30 s. The recovery behavior is characterized by an exponential viscoelastic decay toward a permanent set ε_∞ (dashed black line), which quantifies the residual viscoplastic deformation. The inset table provides the calibrated constitutive parameters (E_1, E_2, γ, σ_y) and the resulting relaxation time τ=16.0 s, showing the balance between elastic driving forces and viscoplastic dissipation.

Table S1. Parameter Table.

Parameter	Symbol	Value	Unit	From Experiment [x]	From Literature [x]	Fitted [f] or Assumed [a]	Variation range in Sensitivity Analysis	Parameter Calibration [x]
Density PLA	ρ_{PLA}	1250	[kg/m^3]	x	-	-	-	-
Density PCL	ρ_{PCL}	1150	[kg/m^3]	x	-	-	-	-
Specific heat c_p - PLA	$c_{p,\,PLA}$	1800	[J/kg·K]	x	-	-	-	-
Specific heat c_p - PCL	$c_{p,\,PCL}$	2000	[J/kg·K]	x	-	-	-	-
Glassy modulus - PLA	$E_{glassy,PLA}$	3x10^9	[Pa]	-	x	-	-	-
Glassy modulus - PCL	$E_{glassy,PCL}$	0.4x10^9	[Pa]	-	x	-	-	-
Rubbery modulus - PLA	$E_{rubbery,PLA}$	4.5x10^7	[Pa]	-	x	-	-	-
Rubbery modulus - PCL	$E_{rubbery,PCL}$	1.5x10^7	[Pa]	-	x	-	-	-
Glass trans. temp. PLA	$T_{g,PLA}$	60	[°C]	x	-	-	-	-
Glass trans. temp. PCL	$T_{g,PCL}$	-60	[°C]	x	-	-	-	-
Effective glass trans.	$T_{g,eff}$	54.15	[°C]	-	-	a	$\pm$5	x
Degree of crystallinity PLA	χ_{PLA}	14.3	[%]	x	-	-	$\pm$30%	-
Degree of crystallinity PCL	χ_{PCL}	42.6	[%]	x	-	-	$\pm$30%	-
Crystalline Modulus PLA	$E_{c,PLA}$	4.31	[GPa]	x	-	-	$\pm$5%, $\pm$10%	-
Amorphous Modulus PLA	$E_{a,PLA}$	266.4	[MPa]	x	-	-	$\pm$10%	-
Crystalline Modulus PCL	$E_{c,PCL}$	154	[MPa]	x	-	-	$\pm$5%	-
Amorphous Modulus PCL	$E_{a,PCL}$	76.7	[MPa]	x	-	-	$\pm$10%	-
PCL content	ω_{PCL}	5	[WT%]	x	-	-	1, 15	-
Transition sharpness	ΔT_{ts}	1.35	[°C]	-	-	a	$\pm$3	x
Conv. heat transf. coeff.	h	200	[W/m^{2}K]	-	x	-	$\pm$100	-
Bath temperature	T_{bath}	60	[°C]	x	-	-	$\pm$15	-
Instan. elastic resp. time	τ_0	0.5	[s]	-	-	a	-	x
Viscoel. coupling coeff.	v_{cc}	2	[/]	-	-	a	-	x
Thermal response time	τ_{rt}	2.6	[s]	-	-	a	$\pm$1	x
Smoothing parameter	$\beta(\chi_{blend})$	1.5	[/]	-	-	a	0.5, 3	x
Thermal onset transition	$\Delta T(\chi_{blend})$	3	[°C]	-	-	a	$\pm$2	x
Therm. kinetic sensitivity	β_{th}	2.002	[deg·s^{-}[illegible]°C^{-}[illegible]]	-	-	f	-	-
Minimum response time	τ_{min}	1.05	[s]	-	-	a	0.5, 2	x
Thermal expansion coeff.	α	1.2·10^{-4}	[K^{-1}]	-	-	a	-	x
Initial temperature	T_0	25	[°C]	x	-	-	15, 40	-
Test temperature	T_{test}	25	[°C]	-	-	a	-	x
Temperature of reference	T_{ref}	0	[°C]	-	-	a	-	x
Fixing ratio	R_f	96.7	[%]	x	-	-	90,100	-
Reinfor. effic. crystal. phase	γ	10	[/]	-	-	a	-	x
